# Acyclic Triterpenoids from *Alpinia katsumadai* Inhibit IL-6-Induced STAT3 Activation

**DOI:** 10.3390/molecules22101611

**Published:** 2017-09-25

**Authors:** Hyun-Jae Jang, Seung-Jae Lee, Soyoung Lee, Kyungsook Jung, Seung Woong Lee, Mun-Chual Rho

**Affiliations:** Immunoregulatory Material Research Center, Korea Research Institute of Bioscience and Biotechnology, 181 Ipsin-gil, Jeongeup-si, Jeonbuk 56212, Korea; water815@kribb.re.kr (H.-J.J.); seung99@kribb.re.kr (S.-J.L.); sylee@kribb.re.kr (S.L.); jungks@kribb.re.kr (K.J.)

**Keywords:** *Alpinia katsumadai*, acyclic triterpenoids, IL-6, STAT3, inflammation

## Abstract

The seeds of *Alpinia katsumadai* yielded two new acyclic triterpenoids, 2,3,6,22,23-pentahydroxy-2,6,11,15,19,23-hexamethyl-tetracosa-7,10,14,18-tetraene (**3**) and 2,3,6,22,23-pentahydroxy-2,10,15,19,23-hexamethyl-7-methylenetetracosa-10,14,18-triene (**4**), as well as two known compounds, 2,3,22,23-tertrahydroxy-2,6,10,15,19,23-hexamethyl-tetracosa-6,10,14,18-tetraene (**1**) and 2,3,5,22,23-pentahydroxy-2,6,10,15,19,23-hexamethyl-tetracosa-6,10,14,18-tetraene (**2**). The absolute configurations of **2** and **3**, which were determined by means of a modified Mosher’s method, are suggested as (**3*R***; **5*S***; **22*R***) and (**3*R***; **22*R***), respectively. Compounds **1**–**4** inhibited IL-6-induced JAK2/STAT3 activity in a dose-dependent fashion, with IC_50_ values of 0.67, 0.71, 2.18, and 2.99 μM. Moreover, IL-6-stimulated phosphorylation of STAT3 was significantly suppressed in U266 cells by the administration of *A. katsumadai* EtOH extract and Compounds **1** and **2**. These results suggest that major phytochemicals, Compounds **1** and **2**, obtained from *A. katsumadai* may be useful candidates for designing new IL-6 inhibitors as anti-inflammatory agents.

## 1. Introduction

Interleukin-6 (IL-6) is a pro-inflammatory cytokine that is secreted by immune and inflammatory-related cells (T cells and macrophages) to stimulate responses to viral infection, trauma, and other tissue damage [1,2]. Several studies have demonstrated that overproduction of IL-6 is relevant to many human diseases, such as cancer cachexia [3], rheumatoid arthritis [4], atherosclerosis [5], diabetes [6], and multiple myeloma [7]. IL-6 also impairs the endothelium-dependent dilatation of human veins in vivo [8]. Therefore, IL-6 is implicated in the pathogenesis of immune and inflammatory diseases, and blocking IL-6 would be an effective treatment for many of these human diseases. The seeds of *Alpinia katsumadai* Hayata (Zingiberaceae) are widely utilized as a traditional Chinese herbal medicine to treat inflammatory and digestive diseases [9]. The seeds contain a variety of major constituents, including diarylheptanoids [10,11,12], monoterpenes [13,14], sesquiterpenoids [15], flavonoids [16], and chalcones [15]. Additionally, the extracts and compounds isolated from this plant exhibit various biological properties, including antiemetic [12], antiproliferative [17], antiviral [18,19], antiasthmatic [20], antiseptic [21], and cytoprotective effects [22]. We have searched for IL-6 inhibitors from natural sources, and the EtOH extract of the seeds of *A. katsumadai* (AKEE) exhibited potent inhibitory effects on IL-6-induced STAT3 (signal transducer and activator of transcription 3) activity (see Table S1 in Supplementary Materials). Herein, we report the isolation and structural elucidation of new acyclic triterpenoid derivatives (**3** and **4**), and describe the biological properties of Compounds **1**–**4**.

## 2. Results and Discussion

### 2.1. Isolation of Compounds

The EtOH extract was suspended in H_2_O and partitioned with CHCl_3_. CHCl_3_-soluble materials, including active substances, were fractionated via open-column chromatography on silica gel and ODS (octadecylsilanized silica gel, C_18_) and subjected to semi-preparative HPLC to yield two known acyclic triterpenoids (**1** and **2**) and two new compounds (**3** and **4**).

### 2.2. Determination of the Acyclic Triterpenoids Structure

**Figure 1:**
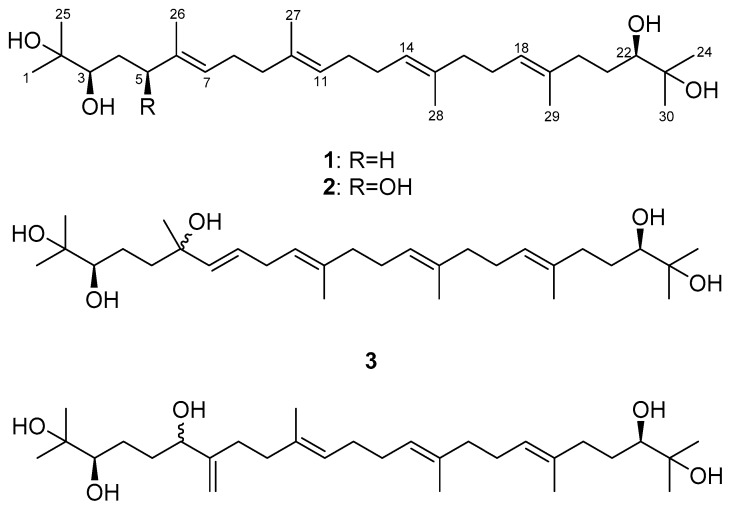
Chemical structure of Compounds **1**–**4**.

Compound **1** was obtained as a yellow oil with [α]${}_{D}^{20}$ +18.1 (CHCl_3_, *c* 1.0). It exhibited a sodium adduct ion peak at *m*/*z* 501 [M + Na]^+^ in the ESIMS and a molecular formula of C_30_H_54_O_4_. The ^13^C-NMR spectrum of **1** contained 15 peaks, which was half the number predicted from the ESIMS spectral data. A symmetrical structure with 30 carbons was suggested. The ^1^H-NMR spectrum of **1** showed signals due to 10 methylene groups (δ_H_ 1.40, 1.58, 1.99~2.08 and 2.23), four methyl groups (δ_H_ 1.59 and 1.61), and four olefinic groups (δ_H_ 5.13 and 5.18). Two methine groups (δ_H_ 3.36, dd, *J* = 10.5, 2.0 Hz) attached to hydroxy groups, and four terminal methyl groups (δ_H_ 1.15 and 1.19) were also observed. In addition, signals at δ_C_ 73.0 and 78.2, attributed to oxygenated carbon, indicated the presence of a hydroxy group in the ^13^C-NMR spectrum of **1**. The connectivity of proton and carbon atoms was assigned based on ^1^H, ^13^C and HMQC spectra. Therefore, the structure of **1** was identified as 2,3,22,23-tetrahydroxy-2,6,10,15,19,23-hexamethyl-tetracosa-6,10,14,18-tetraene by spectroscopic methods (^1^H-, ^13^C-NMR and MS) and by comparing the data with previously reported values (Figure 1) [23]. The absolute configuration was identified as **3*R***, **22*R*** by comparison of the optical rotation value and ^1^H-NMR spectra in previous reports [24].

**Figure 2:**
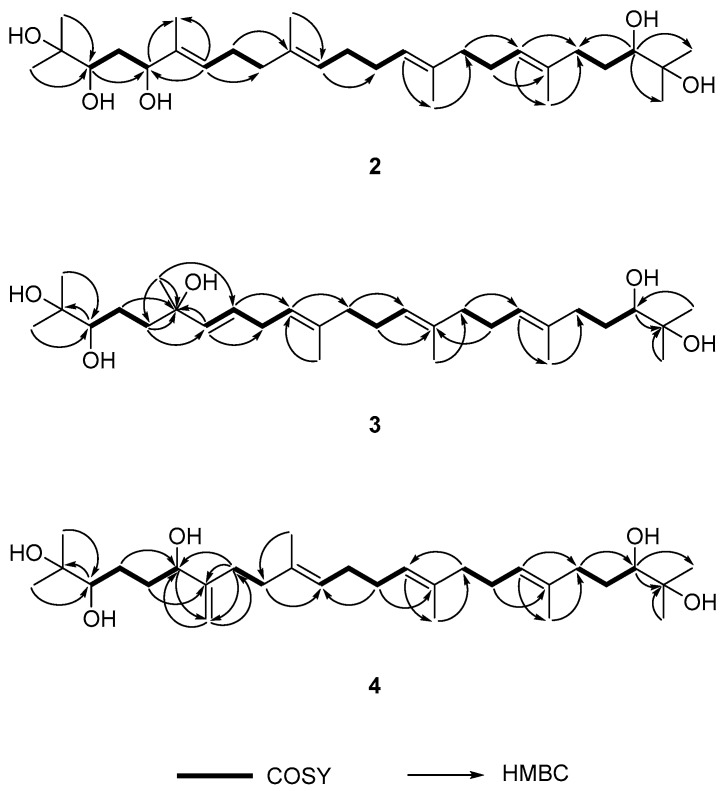
Key COSY and HBMC correlations of Compounds **2**–**4**.

Compound **2** was isolated as a yellow oil with [α]${}_{D}^{20}$ +4.8 (CHCl_3_, *c* 1.0). It displayed a peak at *m*/*z* 493.3897 in the spectrum obtained by high-resolution electrospray ionization mass spectrometry (HRESIMS) corresponding to [M − H]^−^ (calcd. 493.3893), indicating a molecular formula of C_30_H_54_O_5_. In the ^1^H-NMR spectrum of **2** (Table 1), four olefinic protons were observed at δ_H_ 5.14 (2H, m, H-11, H-14), 5.19 (1H, t, *J* = 6.4 Hz, H-18), and 5.43 (1H, t, *J* = 6.4 Hz, H-7), which indicated that **2** has a more asymmetrical structure than **1**. In addition, six allylic methylene groups at δ_H_ 1.41, 1.59, 2.02, 2.05, 2.10, and 2.13 (each 2H, m, H_2_-12, 13, 16, 9, 17, 8), four methyl groups at δ_H_ 1.60, 1.61, 1.62 and 1.63 (each 3H, s, H_3_-29, 28, 27, 26), and two methylene groups at δ_H_ 2.09 and 2.23 (each 2H, m, H_2_-21 and 20) were observed. A methylene group between two hydroxy groups was observed at δ_H_ 1.63 (2H, m, H_2_-4). Additionally, the signals of three methine groups at δ_H_ 3.35 (1H, d, *J* = 10.4 Hz, H-22), 3.62 (1H, m, H-3), and 4.26 (1H, dd, *J* = 7.6, 4.8 Hz, H-5) and four terminal methyl groups at δ_H_ 1.15, 1.17, 1.19, and 1.20 (each 3H, s, H_3_-30, 25, 24, 1) were observed. The ^13^C-NMR spectrum of **2** (Table 1) revealed the presence of 30 carbons, and the connectivity of the proton and carbon atoms was elucidated via DEPT and HMQC analyses. In the ^13^C-NMR spectrum, the signals at δ_C_ 78.5, 78.6, and 78.9 were attributed to methine carbon and suggested the presence of three hydroxy groups. In the HMBC experiment (Figure 2), long-range couplings were observed from H-7 (δ_H_ 5.43) to C-5 (δ_C_ 78.6), C-9 (δ_C_ 39.4) and C-26 (δ_C_ 11.9), from H-5 (δ_H_ 4.26) to C-3 (δ_C_ 78.5), C-7 (δ_C_ 126.6) and C-26 (δ_C_ 11.9), and from H-3 (δ_H_ 3.62) to C-5 (δ_C_ 78.6). The ^1^H-^1^H COSY spectrum of **2** showed correlated proton signals of H_2_-4 (δ_H_ 1.63) with H-3 (δ_H_ 3.62) and H-5 (δ_H_ 4.26). Therefore, we confirmed that the hydroxy group was attached at the C-5 of **2**. We also observed long-range correlations from H-18 (δ_H_ 5.43) to C-20 (δ_C_ 37.0) and C-29 (δ_C_ 16.2) and from H-22 (δ_H_ 3.35) to C-20 (δ_C_ 37.0), C-24 (δ_C_ 23.5), and C-30 (δ_C_ 26.6) in the HMBC spectrum of **2** (Figure 2). In addition, correlated proton signals at H_2_-8 (δ_H_ 2.13), H-7 (δ_H_ 5.43), H_2_-17 (δ_H_ 2.10), and H-18 (δ_H_ 5.19) were observed in the ^1^H-^1^H COSY spectrum of **2**. Based on these results, **2** was identified as the acyclic triterpenoid, 2,3,5,22,23-pentahydroxy-2,6,10,15,19,23-hexamethyl-tetracosa-6,10,14,18-tetraene (Figure 1) [25].

Compound **3** was isolated as a yellow oil with [α]${}_{D}^{20}$ −0.4 (CHCl_3_, *c* 0.1). It displayed a peak at *m*/*z* 517.3861 in the HRESIMS corresponding to [M + Na]^+^ (calcd. 517.3863), indicating a molecular formula of C_30_H_54_O_5_. Comparison of the ^1^H-NMR data with those of **2** (Table 1) indicated that **3** had two downfield-shifted olefinic protons at δ_H_ 5.50 (1H, dd, *J* = 15.6, 1.2 Hz, H-7) and 5.58 (1H, dt, *J* = 15.6, 6.0 Hz, H-8). The downfield effect indicates the presence of an electron-withdrawing group around these protons. In addition, six methylene groups at δ_H_ 1.58, 2.03, 2.10, 2.10, and 2.74 (each 2H, m, H_2_-13, 16, 12, 17, 9), 2.02 and 2.22 (each 1H, m, H_2_-20a, 20b) attached to an olefinic group, two oxymethine groups at δ_H_ 3.35 (1H, dd, *J* =10.8, 1.8 Hz, H-3, H-22), and four terminal methyl groups at δ_H_ 1.15, 1.15, 1.19, and 1.19 (each 3H, s, H_3_-24, 25, 1, 30) were detected. The ^13^C-NMR spectrum of **3** (Table 1) revealed the presence of 30 carbons, and the connectivity of the proton and carbon atoms was elucidated via DEPT and HMQC analyses. In the ^13^C-NMR spectrum, the three oxygenated quaternary carbons C-6 (δ_C_ 73.0), C-2 (δ_C_ 73.1), and C-23 (δ_C_ 73.1) were elucidated via DEPT and HMQC. Detailed 2D correlation analysis identified long-range coupling from H-7 (δ_H_ 5.50) to C-6 (δ_C_ 73.0), C-8 (δ_C_ 126.7), and C-9 (δ_C_ 30.8), from H_2_-5 (δ_H_ 1.55) to C-4 (δ_C_ 22.9), C-6 (δ_C_ 73.0), and C-7 (δ_C_ 136.7), and from H_3_-26 (δ_H_ 1.26) to C-5 (δ_C_ 42.4), C-6 (δ_C_ 73.0), and C-7 (δ_C_ 136.7) (Figure 2). The ^1^H-^1^H COSY spectrum of **3** was from the H_2_-9 (δ_H_ 2.74) *sp*^3^ methylene proton to H-8 (δ_H_ 5.58) and H-10 (δ_H_ 5.14). Therefore, the structure of **3** had a newly attached hydroxy group at the C-6 position. This hydroxy group influenced the shift of the double bond position from H-6 to H-7. Based on these data, **3** was identified as a new acyclic triterpenoid, 2,3,6,22,23-pentahydroxy-2,6,11,15,19,23-hexamethyl-tetracosa-7,10,14,18-tetraene (Figure 1).

Compound **4** was isolated as a yellow oil with [α]${}_{D}^{20}$ +13.3 (CHCl_3_, *c* 0.1). It displayed a peak at *m*/*z* 493.3898 in the HRESIMS corresponding to [M − H]^−^ (calcd. 493.3898), indicating a molecular formula of C_30_H_54_O_5._ Comparison of the ^1^H-NMR data with those of **3** (Table 1) indicated that **4** had three olefinic groups at δ_H_ 5.14 (1H, q, *J* = 6.6 Hz, H-14, -18), and 5.22 (1H, t, *J* = 6.6 Hz, H-11) and two terminal olefinic protons at δ_H_ 4.87 (1H, brs, H_2_-26a) and 5.05 (1H, brs, H_2_-26b), which were supported by the DEPT and COSY spectra. In addition, two methylene group signals between two hydroxy groups were observed at δ_H_ 1.58 (2H, m, H_2_-4) and 2.01 (2H, m, H_2_-5), three oxymethine groups were observed at δ_H_ 3.34 (1H, d, *J* = 9.6 Hz, H-3, -22), and 4.09 (1H, m, H-6), and four terminal methyl groups were observed at δ_H_ 1.15, 1.15, 1.19, and 1.19 (each 3H, s, H_3_-1, 30, 24, 25). In the ^13^C-NMR spectrum (Table 1), three oxymethine carbons, C-6 (δ_C_ 75.1), C-3 (δ_C_ 78.1), and C-22 (δ_C_ 78.1), were elucidated via DEPT and HMQC analyses. Detailed 2D correlation analysis between C-6 and terminal olefinic protons revealed long-range couplings from H_2_-5 (δ_H_ 2.01) to C-6 (δ_C_ 75.1) and C-7 (δ_C_ 151.3), from H-6 (δ_H_ 4.09) to C-5 (δ_C_ 24.3) and C-26 (δ_C_ 109.9), from H_2_-8 (δ_H_ 2.19) to C-6 (δ_C_ 75.1), C-26 (δ_C_ 109.9), and C-7 (δ_C_ 151.3), and from H_2_-26 (δ_H_ 4.87 and 5.05) to C-6 (δ_C_ 75.1) and C-8 (δ_C_ 31.0). Therefore, Compound **4** had a secondary alcohol group (C-6) instead of the methyl group at the C-6 of **3**. This hydroxy group influenced the movement of the double bond position, and terminal olefinic protons appeared at the C-7 position. Based on these results, Compound **4** was identified as a new acyclic triterpenoid, 2,3,6,22,23-pentahydroxy-2,10,15,19,23-hexamethyl-7-methylenetetracosa-10,14,18-triene.

**Figure 3:**
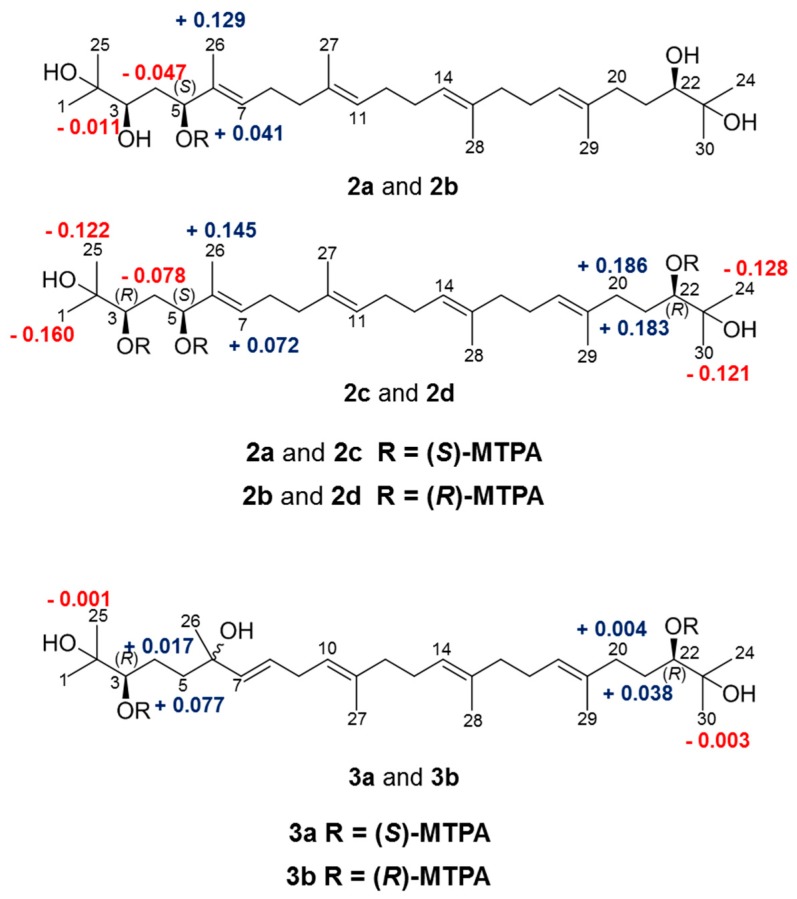
Δδ (δ*_S_ −* δ*_R_*) values in ppm for the MTPA esters of **2** and **3**.

We applied the modified Mosher’s method to determine the absolute configuration of the three chiral centers at C-3, C-5, and C-22 in **2**, and corresponding to the Δδ_H_ pattern for the stereochemistry of secondary diols reported by Freire et al. [26,27,28]. The mono- or tris-(*S*)-MTPA esters (**2a**, **2c**) and -(*R*)-MTPA esters (**2b**, **2d**) were prepared by treating **2** with (*R*)-, and (*S*)-MTPA chloride, respectively. The difference in the chemical shift values (Δδ_H_ = δ*_S_* − δ*_R_*) of (*S*)-MTPA (**2a**, **2c**), and (*R*)-MTPA ester derivatives (**2b**, **2d**) was calculated to assign the absolute configuration of **2** (Figure 3). Based on the results summarized in Figure 3, the absolute configurations of C-3, C-5, and C-22 in **2** were determined as *R*, *S* and *R*, respectively. To elucidate absolute configuration of Compound **3**, a pair of MTPA esters (**3a** and **3b**) were prepared in the same manner. As shown in Figure 3, the absolute configurations of C-3 and C-22 in **3** were assigned as *R* and *R*, respectively. Unfortunately, we were unable to prepare the relevant MTPA ester derivatives to determine the absolute configuration of **4**, and further studies are required to elucidate stereostructure of acyclic triterpenoid (**4**).

### 2.3. IL-6/STAT3 Inhibitory Effects of Acyclic Triterpenoids ***1**–**4***

**Figure 4:**
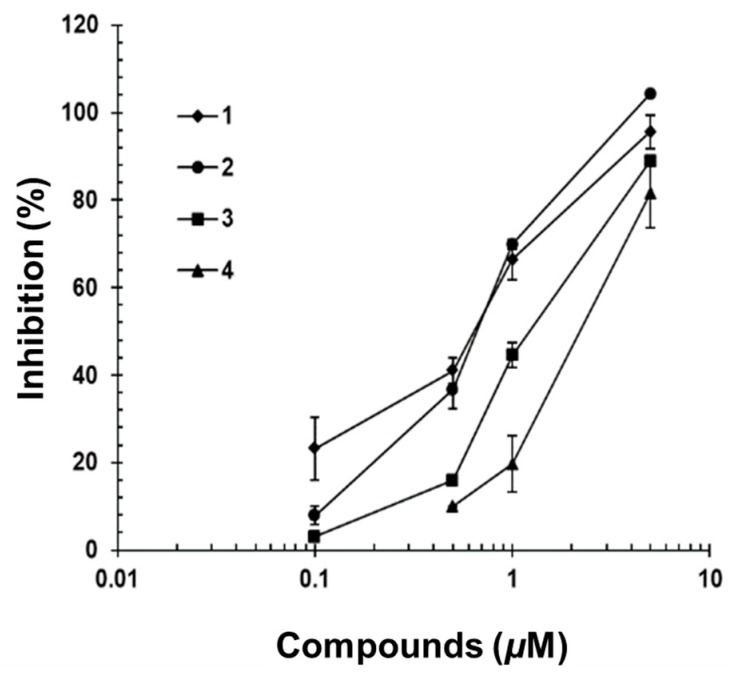
Inhibitory effects of **1**–**4** on IL-6/STAT3 transcriptional activity in Hep3B cells. The pSTAT3-inducible luciferase activity was measured by luciferase assay. Three independent experiments were performed, and the results are presented as the means ± standard error (S.E.).

Compounds **1**–**4** were tested for their inhibitory effects on STAT3-dependent luciferase activity induced by IL-6. Hep3B cells stably transformed with the pStat3-Luc plasmid were stimulated with IL-6 (10 ng/mL) for 12 h in the presence or absence of Compounds **1**–**4**, and STAT3-dependent promoter activity was measured [29]. Compounds **1**–**4** inhibited IL-6-induced STAT3 activity in a dose-dependent fashion, with IC_50_ values of 0.67, 0.71, 2.18 and 2.99 μM (Figure 4). These four acyclic triterpenoids showed more potent inhibitory activity on STAT3-dependent luciferase activity than that of genistein as the positive control (IC_50_ value, 15.0 µM in this assay system) [29]. Additionally, Compounds **1**–**4** were non-cytotoxic at the IC_50_ dose indicated in this study (see Figure S34 in Supplementary Materials).

**Figure 5:**
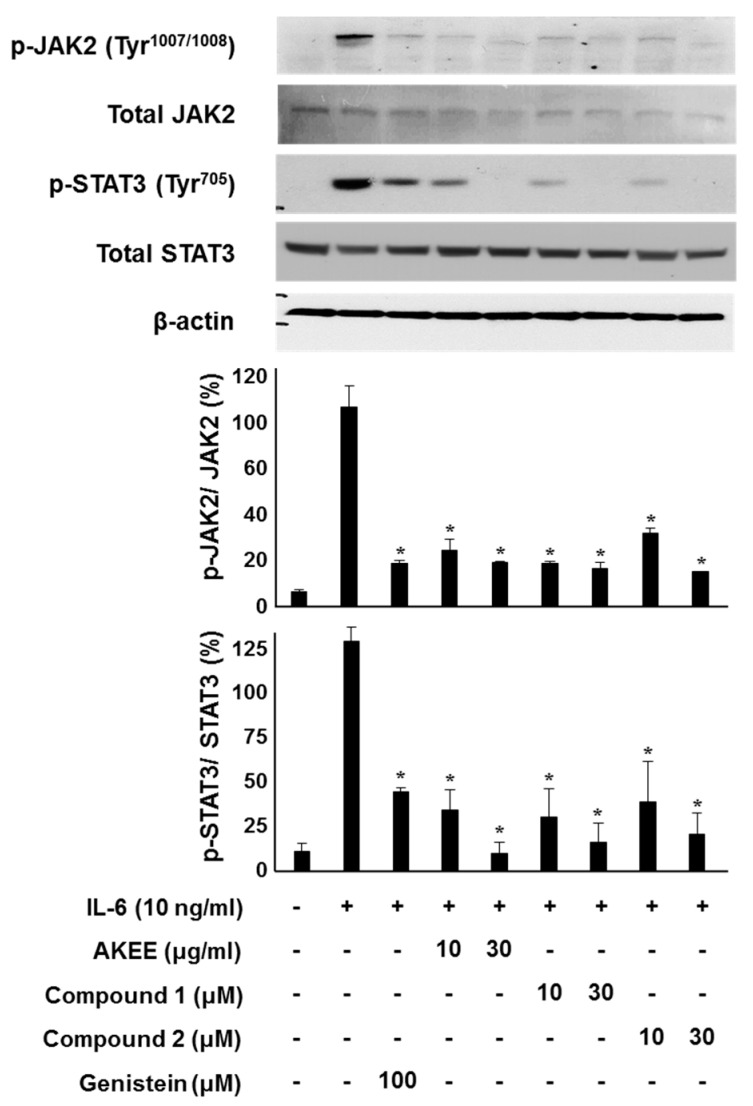
Inhibitory effects of *A. katsumadai* EtOH extract (AKEE) and its compounds (**1** and **2**) on IL-6-induced JAK2 and STAT3 phosphorylation in U266 cells. Cells were pre-treated with samples for 1 h at the indicated concentrations and were then treated with IL-6 (10 ng/mL) for 20 min. Phosphorylated JAK2 and STAT3 was analyzed by Western blotting. The ratios of p-JAK2 or p-STAT3/β-actin were measured using ImageJ software (1.48v, US National Institutes of Health, Bethesda, MD, USA). The data were analyzed by *t*-test compared with the IL-6-induced group, and an asterisk (*) indicates significant difference (*p* < 0.05).

The IL-6-induced JAK2/STAT3 signaling pathway plays a positive role in inflammation and neoplasia [30]. The phosphorylation of JAK2 and STAT3 leads to dimerization of STAT3 and translocation to the nucleus, where it transcribes pro-inflammatory cytokine genes, such as IFN-γ, IL-17, and IL-1β [31]. To determine whether the inhibitory effects on IL-6/STAT3 are dependent on JAK2/STAT3 phosphorylation, we evaluated the protein level by Western blotting analysis. As shown in Figure 5, *A. katsumadai* EtOH extract and Compounds **1** and **2** inhibited IL-6-stimulated phosphorylation of JAK2 and STAT3 in a concentrations-dependent manner. In particular, Compounds **1** and **2**, which were major constituents of the *A. katsumadai* EtOH extract (see Figure S35 in Supplementary Materials), were significantly responsible for inhibiting IL-6/STAT3 activation.

## 3. Materials and Methods

### 3.1. General Experimental Procedures

^1^H-NMR (300, 500 and 600 MHz), ^13^C-NMR (75, 125 and 150 MHz), HMQC, and HMBC spectra were obtained on a Varian Unity 300, Bruker Biospin Avance 500, and JEOL JNM-ECA600 spectrometer, with CDCl_3_ as a solvent. ESI-MS was conducted using a Shimadzu LCMS-IT-TOF mass spectrometer. Optical rotations were determined on a JASCO DIP-370 polarimeter. The HPLC system consisted of a Hitachi L-2130 pump, Hitachi UV detector L-2400, and Capcell Pak C18 column (20 × 250 mm, Shiseido, Tokyo, Japan). Reversed-phase column chromatography was conducted using RP-C_18_ silica gel (ODS-A, 250 × 20 mm, YMC Co. Ltd., Kyoto, Japan), and silica gel column chromatography was conducted using Kieselgel 60 (70–230 and 200–400 mesh, Merck, Darmstadt, Germany). TLC was conducted using Kieselgel 60 F_254_ plates (Merck).

### 3.2. Extraction and Isolation

The seeds of *A. katsumadai* were purchased at a herbal market in Daejeon, Korea. The authenticity of the plants was confirmed by Prof. Y. H. Kim, at the College of Pharmacy of Chungnam National University (Daejeon, Korea). A voucher specimen (PBC-386A) was deposited in the Korea Plant Extract Bank at the Korea Research Institute of Bioscience and Biotechnology. Dried seeds of *A. katsumadai* (1.8 kg) were extracted with EtOH (10 L) for 7 days at room temperature. The ethanol extract was evaporated in vacuo to yield a residue (180 g). The residue was suspended in distilled H_2_O (3 L) and extracted with CHCl_3_ (10 L). The CHCl_3_-soluble components were then evaporated in vacuo, and the resulting extract (85 g) was subjected to silica gel (Kieselgel 60, 230–400 mesh, 150 g, Merck, Darmstadt, Germany) column chromatography using a gradient of CHCl_3_–CH_3_OH (100:0, 90:1, 70:1, 50:1, 30:1, 15:1, 5:1 and 1:1; each 3 L, *v*/*v*) as eluent to yield 22 fractions (F1-22) on the TLC profile. F8 (2.4 g) was subjected to reverse-phase column chromatography (100 g) and was eluted with CH_3_OH–H_2_O (60:1, 70:1, 80:1, 90:1 and 100:0; each 2 L, *v*/*v*), to yield six sub-fractions (F8-1, -6) based on the TLC profile. F8-3 (1.0 g) was separated by semi-preparative HPLC (YMC ODS–H80, flow rate: 6 mL/min) eluted with CH_3_CN–H_2_O (80:1, *v*/*v*) to yield **1** (300 mg, *t*_R_ 40 min). F9 (700 mg) was subjected to reverse-phase column chromatography (100 g) eluted with CH_3_OH–H_2_O (50:1, 60:1, 70:1, 80:1, 90:1, and 100:0; each 2 L, *v*/*v*) to yield seven sub-fractions (F9-1, -7). F9-3 (300 mg) was successively separated by preparative HPLC (CH_3_CN–H_2_O, 70:1, *v*/*v*) to yield **2** (100 mg, *t*_R_ 30 min). F16 (8.4 g) was subjected to reverse-phase column chromatography eluted with CH_3_OH–H_2_O (2:3, 1:1, 3:2, 7:3, 4:1, 9:1, and 100:0; each 500 m L, *v*/*v*), to yield seven sub-fractions (F16-1, -7). F16-4 was subjected to reverse-phase column chromatography eluted with CH_3_CN–H_2_O (2:3, 1:1, 3:2, 7:3, 4:1, 9:1, and 100:0; each 300 mL, *v*/*v*) to yield seven sub-fractions (F16-4-1, -7). F16-4-6 (538 mg) was eluted with CH_3_OH–H_2_O (4:1, *v*/*v*) and was subjected to further separation by preparative HPLC (Capcell Pak C_18_, flow rate: 5 mL/min) to obtain five fractions. F16-4-6-3 was purified by semi-preparative HPLC (CH_3_CN–H_2_O, 11:9, flow rate: 5 mL/min) to yield **3** (30 mg, *t*_R_ 38 min). F16-4-6-5 was purified by preparative HPLC (CH_3_CN–H_2_O, 3:2, flow rate: 5 mL/min) to yield **4** (32 mg, *t*_R_ 45 min).

*2,3,22,23-Tertrahydroxy-2,6,10,15,19,23-hexamethyl-tetracosa-6,10,14,18-tetraene* (**1**). Yellow oil; C_30_H_54_O_4_; [α]${}_{D}^{20}$ +18.1 (*c* 1.0, CHCl_3_); IR (neat) ν_max_ 3400, 2950 cm^−1^; ESI-MS: *m*/*z* 501 [M + Na]^+^; ^1^H-NMR (300 MHz, CDCl_3_) δ_H_ 5.18 (2H, m, H-7, H-18), 5.13 (2H, m, H-11, H-14), 3.36 (2H, dd, *J* = 2.0, 10.5 Hz, H-3, H-22), 2.23 (2H, m, H_2_-5a, H_2_-20a), 2.08 (2H, m, H-8, H-17), 2.06 (2H, m, H_2_-5b, H_2_-20b), 2.01 (2H, m, H_2_-9, H_2_-16), 1.99 (2H, m, H_2_-12, H_2_-13), 1.59 (3H, s, H_3_-27, H_3_-28), 1.58, (2H, m, H_2_-4a, H_2_-21a), 1.61 (3H, s, H_3_-26, H_3_-29), 1.40 (2H, m, H_2_-4b, H_2_-21b), 1.19 (3H, s, H_3_-1, H_3_-24), 1.15 (3H, s, H_3_-25, H_3_-30); ^13^C-NMR (75 MHz, CDCl_3_) δ_C_ 15.88 (C-27, C-28), 15.95 (C-26, C-29), 23.23 (C-25, C-30), 26.36 (C-1, C-24), 26.47 (C-8, C-17), 28.17 (C-9, C-16), 29.64 (C-4, C-21), 36.77 (C-5, C-20), 39.61 (C-12, C-13), 72.99 (C-2, C-23), 78.23 (C-3, C-22), 124.40 (C-11, C-14), 125.01 (C-7, C-18), 134.79 (C-10, C-15), 134.91 (C-6, C-19).

*2,3,5,22,23-Pentahydroxy-2,6,10,15,19,23-hexamethyl-tetracosa-6,10,14,18-tetraene* (**2**). Yellow oil; C_30_H_54_O_5_; [α]${}_{D}^{20}$ +4.8 (*c* 1.0, CHCl_3_); IR (neat): ν_max_ 3400, 2900 cm^−1^; UV(MeOH) λ_max_ (logε) nm: 210 (2.51); ^1^H-NMR (500 MHz, CDCl_3_) and ^13^C-NMR (125 MHz, CDCl_3_) spectra, see Table 1; HRESI-MS: *m*/*z* 493.3897 [M − H]^−^ (calcd. for C_30_H_53_O_5_, 493.3893).

*2,3,6,22,23-Pentahydroxy-2,6,11,15,19,23-hexamethyl-tetracosa-7,10,14,18-tetraene* (**3**). Yellow oil; C_30_H_54_O_5_; [α]${}_{D}^{20}$ −0.4 (*c* 0.1, CHCl_3_); IR (MeOH): ν_max_ 3300, 2950, 1450, 1050 cm^−1^; UV(MeOH) λ_max_ (logε) nm: 205 (2.42); ^1^H-NMR (600 MHz, CDCl_3_) and ^13^C-NMR (150 MHz, CDCl_3_) spectra, see Table 1; HRESI-MS: *m*/*z* 517.3861 [M + Na]^+^ (calcd. for C_30_H_54_O_5_Na, 517.3863).

*2,3,6,22,23-Pentahydroxy-2,10,15,19,23-hexamethyl-7-methylenetetracosa-10,14,18-triene* (**4**). Yellow oil; C_30_H_54_O_5_; [α]${}_{D}^{20}$ +13.3 (*c* 0.1, CHCl_3_); IR (MeOH): ν_max_ 3300, 2950, 1050 cm^−1^; UV(MeOH) λ_max_ (logε) nm: 205 (2.34); ^1^H-NMR (600 MHz, CDCl_3_) and ^13^C-NMR (150 MHz, CDCl_3_) spectra, see Table 1; HRESI-MS: *m*/*z* 493.3899 [M − H]^−^ (calcd. for C_30_H_53_O_5_, 493.3898).

### 3.3. (R)- and (S)-MTPA Ester of Compounds ***2**–**3***

Compounds **2**–**3** (4 mg) were dissolved in pyridine-*d*_5_ (10 mL), and (*R*)- or (*S*)-MTPA-Cl (10 μL) and DMAP (2 mg) were added. The mixture was transferred vials under an N_2_ gas stream. The each vials were incubated in a water bath for 4 h (40 °C). The reactions afforded (*S*)- or (*R*)-MTPA ester derivatives **2*S***, **3*S***, and **2*R***, **3*R***, and each residues were purified by preparative HPLC using a C_18_ column (Phenomenex kinetex C_18_, 150 × 21.0 mm), eluting with a gradient solvent system composed of H_2_O/CH_3_OH (40:60 → 0:100, *v*/*v*). The ^1^H-NMR spectra of each derivative were obtained from the reaction.

*5-Mono-MTPA Ester of* **2** (**2a**). ^1^H-NMR (pyridine-*d*_5_, 600 MHz) δ_H_ 6.41 (1H, dd, *J* = 10.8, 4.2 Hz, H-5), 5.94 (1H, t, *J* = 6.0 Hz, H-7), 5.39 (1H, t, *J* = 7.2, H-18), 5.31 (1H, br s, H-11), 5.26 (1H, br s, H-14), 3.77 (1H, m, H-3), 3.73 (1H, m, H-22), 2.70 (1H, m, H_2_-20a), 2.51 (1H, m, H_2_-4a), 2.40 (5H, m, H_2_-4b, -8a, -13a, -17a, -20b), 2.11 (8H, m, H_2_-8b, -9, 12a, -13b, -16, -17b), 1.83 (3H, s, H_3_-26), 1.82 (2H, m, H_2_-21), 1.71, (3H, s, H_3_-27), 1.66, (3H, s, H_3_-28), 1.62 (3H, s, H_3_-29), 1.54 (3H, s, H_3_-1), 1.52 (3H, s, H_3_-24), 1.51 (3H, s, H_3_-25), 1.48 (3H, s, H_3_-30).

*5-Mono-MTPA Ester of* **2** (**2b**). ^1^H-NMR (pyridine-*d*_5_, 600 MHz) δ_H_ 6.36 (1H, dd, *J* = 10.2, 4.2 Hz, H-5), 5.90 (1H, t, *J* = 5.4 Hz, H-7), 5.39 (1H, t, *J* = 7.2, H-18), 5.31 (1H, br s, H-11), 5.27 (1H, br s, H-14), 3.78 (2H, m, H-3, -22), 2.71 (1H, m, H_2_-20a), 2.56 (1H, m, H_2_-4a), 2.36 (5H, m, H_2_-4b, -8a, -13a, -17a, -20b), 2.08 (8H, m, H_2_-8b, -9, 12a, -13b, -16, -17b), 1.82 (2H, m, H_2_-21), 1.71 (3H, s, H_3_-26), 1.66, (3H, s, H_3_-27), 1.62, (3H, s, H_3_-28), 1.61 (3H, s, H_3_-29), 1.55 (6H, s, H_3_-1, -24), 1.52 (3H, s, H_3_-25), 1.51 (3H, s, H_3_-30).

*3,5,22-Tris-MTPA Ester of* **2** (**2c**). ^1^H-NMR (pyridine-*d*_5_, 600 MHz) δ_H_ 6.06 (1H, dd, *J* = 11.4, 4.2 Hz, H-5), 6.00 (1H, t, *J* = 6.6 Hz, H-7), 5.53 (1H, d, *J* = 10.2, H-3), 5.39 (1H, d, *J* = 10.2 Hz, H-22), 5.36 (1H, t, *J* = 6.6 Hz, H-18), 5.30 (2H, m, H-11, -14), 2.75 (1H, m, H_2_-20a), 2.36 (1H, m, H_2_-4a), 2.30 (5H, m, H_2_-4b, -8a, -13a, -17a, -20b), 2.18 (8H, m, H_2_-8b, -9, 12a, -13b, -16, -17b), 1.95 (2H, m, H_2_-21), 1.81 (3H, s, H_3_-26), 1.66, (3H, s, H_3_-27), 1.65, (3H, s, H_3_-28), 1.62 (3H, s, H_3_-29), 1.40 (3H, s, H_3_-1), 1.39 (3H, s, H_3_-24), 1.33 (3H, s, H_3_-25), 1.32 (3H, s, H_3_-30).

*3,5,22-Tris-MTPA Ester of* **2** (**2d**). ^1^H-NMR (pyridine-*d*_5_, 600 MHz) δ_H_ 6.36 (1H, dd, *J* = 10.2, 4.2 Hz, H-5), 5.90 (1H, m, H-7), 5.55 (1H, d, *J* = 10.8, H-3), 5.32 (1H, m, H-22), 5.27 (1H, m, H-18), 5.20 (2H, m, H-11, -14), 2.56 (1H, m, H_2_-20a), 2.44 (1H, m, H_2_-4a), 2.38 (5H, m, H_2_-4b, -8a, -13a, -17a, -20b), 2.11 (8H, m, H_2_-8b, -9, 12a, -13b, -16, -17b), 1.77 (2H, m, H_2_-21), 1.67 (3H, s, H_3_-26), 1.62, (3H, s, H_3_-27, -28), 1.60 (3H, s, H_3_-29), 1.56 (3H, s, H_3_-1), 1.51 (3H, s, H_3_-24), 1.45 (3H, s, H_3_-25), 1.44 (3H, s, H_3_-30).

*3,22-Bis-MTPA Ester of* **3** (**3a**). ^1^H-NMR (pyridine-*d*_5_, 600 MHz) δ_H_ 5.90 (1H, m, H-7), 5.87 (1H, m, H-8), 5.70 (1H, m, H-18), 5.52 (1H, m, H-14), 5.45 (2H, m, H-3, -22), 5.38 (1H, m, H-10), 2.90 (2H, m, H_2_-9), 2.56 (1H, m, H_2_-20a), 2.01 (1H, m, H_2_-4a), 1.96 (13H, m, H_2_-8, -9, -12, -13, -16, -17, -20b), 1.88 (2H, m, H_2_-21), 1.83 (2H, m, H_2_-5), 1.70 (3H, s, H_3_-28), 1.66, (3H, s, H_3_-29), 1.62, (3H, s, H_3_-27), 1.54 (3H, s, H_3_-1), 1.53 (3H, s, H_3_-30), 1.52 (3H, s, H_3_-26), 1.51 (3H, s, H_3_-24), 1.50 (3H, s, H_3_-25).

*3,22-Bis-MTPA Ester of* **3** (**3b**). ^1^H-NMR (pyridine-*d*_5_, 600 MHz) δ_H_ 5.89 (1H, m, H-7), 5.86 (1H, m, H-8), 5.72 (1H, m, H-18), 5.53 (1H, m, H-14), 5.44 (2H, m, H-3, -22), 5.38 (1H, m, H-10), 2.90 (2H, m, H_2_-9), 2.55 (1H, m, H_2_-20a), 2.08 (1H, m, H_2_-4a), 1.91 (13H, m, H_2_-8, -9, -12, -13, -16, -17, -20b), 1.80 (2H, m, H_2_-21), 1.79 (2H, m, H_2_-5), 1.70 (3H, s, H_3_-28), 1.66, (3H, s, H_3_-29), 1.62, (3H, s, H_3_-27), 1.53 (3H, s, H_3_-1, -30), 1.52 (3H, s, H_3_-26), 1.51 (3H, s, H_3_-24), 1.50 (3H, s, H_3_-25).

### 3.4. Biological Materials and Cell Culture

Recombinant human IL-6 was purchased from R&D Systems (Minneapolis, MN, USA). Mouse anti-phospho Stat3 (Tyr^705^) IgG was purchased from Calbiochem (Darmstadt, Germany). All reagents, including genistein, were obtained from Sigma-Aldrich Ltd. (St. Louis, MO, USA). Human hepatoma Hep3B and U266 cells were obtained from the American Type Culture Collection (ATCC No. HB-8064 and TIB-196TM, Rockville, MD, USA) and were maintained in DMEM and RPMI1640 media supplemented with 10% fetal bovine serum, 50 U/mL penicillin, and 50 mg/mL streptomycin at 37 °C in a 5% CO_2_ incubator. All cell culture reagents were obtained from GibcoBRL (Life Technologies, Cergy-Pontoise, France).

### 3.5. Luciferase Assay

Hep3B cells stably expressing pStat3-Luc, which were previously established by Chang et al. [32], were seeded onto 96-well culture plates at 2 × 10^4^ cells/well. After 24 h, the cells were starved for 12 h and were then treated with IL-6 (10 ng/mL) with or without compounds for 12 h. The luciferase assay was performed with a Promega kit according to the manufacturer’s protocol (Madison, WI, USA).

### 3.6. Cell Viability

Hep3B cells were seeded at a plating density of 2 × 10^4^ cells/well and were cultured for 24 h to allow them to adhere to the plate. After 24 h, the culture medium was changed to serum-free medium supplemented with samples at the indicated dose. MTT (0.5 mg/mL) was added after a 48 h culture, and 200 μL of DMSO was then added to each well after a 4 h incubation at 37 °C. The absorbance of the samples at 540 nm was measured against a background control using a 96-well plate reader. The percentage of viable cells under each treatment condition was determined relative to the negative control.

### 3.7. Western Blotting Analysis

U266 cells were stimulated with IL-6 (10 ng/mL) for 20 min in the presence or absence of compounds. Western blot analysis was performed to evaluate STAT3 and JAK2 protein expression in the U266 cell line, as described in previous studies [33]. The phosphorylation status of STAT3 and JAK2 was examined using anti-phospho-Stat3 (1:1000), anti-Stat3 (1:1000), anti-phospho-Jak2 (1:1000), and anti-Jak2 (1:1000) antibodies (Cell Signaling, Beverly, MA, USA) and then were incubated with the appropriate horseradish peroxide-conjugated secondary antibody (1:5000) at RT. The optical densities of antibody-specific bands were quantified using ImageJ software.

### 3.8. Statistical Analyses

Data are expressed as the means ± standard error of the mean (S.E.), and the statistical analyses were performed using Student’s *t*-test in Prism 5 software (GraphPad software, San Diego, CA, USA). A probability value of 0.05 (*p* < 0.05) was considered significant.

## 4. Conclusions

In conclusion, four acyclic triterpenoid derivatives (**1**–**4**) were isolated from the EtOH extract of *A. katsumadai*. Compounds **3** and **4** were identified as new acyclic triterpenoids 2,3,6,22,23-pentahydroxy-2,6,11,15,19,23-hexamethyl-tetracosa-7,10,14,18-tetraene and 2,3,6,22,23-pentahydroxy-2,10,15,19,23-hexamethyl-7-methylenetetracosa-10,14,18-triene, respectively. In addition, major constituents, Compounds **1** and **2**, in *A. katsumadai* showed more potent inhibitory activity on IL-6-induced STAT3 activation. Based on our results, these triterpenoids could be useful candidates for designing new IL-6 inhibitors as anti-inflammatory agents.

## Figures and Tables

**Table 1:** ^1^H- and ^13^C-NMR Spectroscopic data of Compounds **2**–**4**.

Position	2 ^a^		3 ^b^		4 ^b^	
	δ_H_ (*J* in Hz)	δ_C_	δ_H_ (*J* in Hz)	δ_C_	δ_H_ (*J* in Hz)	δ_C_
1	1.20, s	24.0	1.19, s	26.4	1.15, s	26.4
2	-	73.3	-	73.1	-	73.0
3	3.62, dd (8.4, 3.6)	78.5	3.35, dd (10.8, 1.8)	48.2	3.34, d (9.6)	78.1
4	1.63, m	36.1	2.03, m	22.9	1.40, 1.58, m	29.5
5	4.26, dd (7.6, 4.8)	78.6	1.55, m	42.4	2.01, m	24.3
6	-	134.9	-	73.0	4.09, m	75.1
7	5.43, t (6.4)	126.6	5.50, dd (15.6, 1.2)	136.7	-	151.3
8	2.13, m	26.6	5.58, dt (15.6, 6.0)	126.7	2.01, 2.19, m	31.0
9	2.05, m	39.4	2.74, t (6.6)	30.8	1.58, 1.65, m	35.3
10	-	135.1	5.14, td (7.2, 1.2)	122.2	-	135.9
11	5.14, m	124.7	-	135.0	5.22, t (6.6)	125.1
12	1.41, m	29.8	2.03, 2.10, m	39.5	1.58, m	29.6
13	1.59, m	39.8	1.40, 1.58, m	29.5	2.10, m	29.4
14	5.14, m	124.9	5.18, t (6.6)	124.9	5.14, q (6.6)	124.3
15	-	135.2	-	135.2	-	135.0
16	2.02, m	28.4	2.03, m	36.8	2.02, m	39.5
17	2.10, m	26.6	2.10, m	26.1	2.01, 2.10, m	26.4
18	5.19, t (6.4)	125.3	5.23, td (6.6, 1.2)	125.5	5.14, q (6.6)	124.9
19	-	137.3	-	135.9	-	135.1
20	2.23, m	37.0	2.02, 2.22, m	36.8	2.03, 2.21, m	36.7
21	2.09, m	26.2	1.58, m	29.6	1.40, 1.58, m	29.5
22	3.35, d (10.4)	78.9	3.35, dd (10.8, 1.8)	78.2	3.34, d (9.6)	78.1
23	-	72.8	-	73.1	-	73.0
24	1.19, s	23.5	1.15, s	23.4	1.19, s	23.4
25	1.17, s	26.4	1.15, s	23.3	1.19, s	23.3
26	1.63, s	11.9	1.26, s	28.1	4.87, 5.05, br s	109.9
27	1.62, s	16.1	1.60, s	16.0	1.60, s	15.9
28	1.61, s	16.2	1.61, s	15.9	1.62, s	16.0
29	1.60, s	16.2	1.61, s	15.9	1.61, s	15.9
30	1.15, s	26.6	1.19, s	26.5	1.15, s	26.4

^a 1^H- and ^13^C-NMR spectra were recorded at 500 and 125 MHz, respectively, in CDCl_3_; ^b 1^H- and ^13^C-NMR spectra were recorded at 600 and 150 MHz, respectively, in CDCl_3_.

## Supplementary Materials

Supplementary Materials are available online.
