# Supplementary material for: Acyclic Triterpenoids from Alpinia katsumadai Inhibit IL-6-Induced STAT3 Activation
Source: Molecules. 2017 Sep 25;22(10):1611. doi: 10.3390/molecules22101611 (PMC6151764; doi:10.3390/molecules22101611)
Supplement: Supplementary file 1 [file molecules-22-01611-s001.pdf]

Supplementary Materials

Acyclic Triterpenoids from *Alpinia katsumadai* Inhibit IL-6-Induced STAT3 Activation

Hyun-Jae Jang, Seung-Jae Lee, Soyoung Lee, Kyungsook Jung, Seung Woong Lee * and Mun-Chual Rho *

Immunoregulatory Material Research Center, Korea Research Institute of Bioscience and Biotechnology, 181 Ipsin-gil, Jeongeup-si, Jeonbuk 56212, Korea; water815@kribb.re.kr (H.-J.J.); seung99@kribb.re.kr (S.-J.L.); sylee@kribb.re.kr (S.L.); jungks@kribb.re.kr (K.J.)

* Correspondence: lswdoc@kribb.re.kr (S.W.L.); rho-m@kribb.re.kr (M.-C.R.); Tel.: +82-63-570-5264 (S.W.L.); +82-63-570-5230 (M.-C.R.); Fax: +82-63-570-5239 (S.W.L. and M.-C.R.)

List of Supplementary Materials

Figure S1. ^{1}H NMR spectrum of 2,3,22,23-tertrahydroxy-2,6,10,15,19,23-hexamethyl-tetracosa-6,10,14,18-tetraene (**1**) (300 MHz in $CDCl_3$)······ ···························4

Figure S2. ^{13}C NMR spectrum of 2,3,22,23-tertrahydroxy-2,6,10,15,19,23-hexamethyl-tetracosa-6,10,14,18-tetraene (**1**) (75 MHz in $CDCl_3$)······· ···························5

Figure S3. DEPT NMR spectrum of 2,3,22,23-tertrahydroxy-2,6,10,15,19,23-hexamethyl-tetracosa-6,10,14,18-tetraene (**1**) (75 MHz in Figure $CDCl_3$)···· ··········6

Figure S4. ^{1}H-^{1}H COSY NMR spectrum of 2,3,22,23-tertrahydroxy-2,6,10,15,19,23-hexamethyl-tetracosa-6,10,14,18-tetraene (**1**)············· ···································7

Figure S5. HMQC NMR spectrum of 2,3,22,23-tertrahydroxy-2,6,10,15,19,23-hexamethyl-tetracosa-6,10,14,18-tetraene (**1**)························ ·································8

Figure S6. HMBC NMR spectrum of 2,3,22,23-tertrahydroxy-2,6,10,15,19,23-hexamethyl-tetracosa-6,10,14,18-tetraene (**1**)························· ·······························9

Figure S7. LRESI-MS spectrum of 2,3,22,23-tertrahydroxy-2,6,10,15,19,23-hexamethyl-tetracosa-6,10,14,18-tetraene (**1**)······························ ···························10

Figure S8. ^{1}H NMR spectrum of 2,3,5,22,23-pentahydroxy-2,6,10,15,19,23-hexamethyl-tetracosa-6,10,14,18-tetraene (**2**) (500 MHz in $CDCl_3$)···· ·····················11

Figure S9. ^{13}C NMR spectrum of 2,3,5,22,23-pentahydroxy-2,6,10,15,19,23-hexamethyl-tetracosa-6,10,14,18-tetraene (**2**) (125 MHz in $CDCl_3$)··· ·····················12

Figure S10. ^{1}H-^{1}H COSY NMR spectrum of 2,3,5,22,23-pentahydroxy-2,6,10,15,19,23-hexamethyl-tetracosa-6,10,14,18-tetraene (**2**)············· ···························13

Figure S11. HMQC NMR spectrum of 2,3,5,22,23-pentahydroxy-2,6,10,15,19,23-hexamethyl-tetracosa-6,10,14,18-tetraene (**2**)··········· ·······································14

Figure S12. HMBC NMR spectrum of 2,3,5,22,23-pentahydroxy-2,6,10,15,19,23-hexamethyl-tetracosa-6,10,14,18-tetraene (**2**) ···15

Figure S13. HRESI-MS spectrum of 2,3,5,22,23-pentahydroxy-2,6,10,15,19,23-hexamethyl-tetracosa-6,10,14,18-tetraene (**2**) ··16

Figure S14. ^{1}H NMR spectrum of 5-mono-(*S*)-MTPA ester of **2** (**2a**) ··17

Figure S15. ^{1}H NMR spectrum of 5-mono-(*R*)-MTPA ester of **2** (**2c**) ··18

Figure S16. ^{1}H NMR spectrum of 3,5,22-tris-(*S*)-MTPA ester of **2** (**2b**) ···19

Figure S17. ^{1}H NMR spectrum of 3,5,22-tris-(*R*)-MTPA ester of **2** (**2d**) ···20

Figure S18. ^{1}H NMR spectrum of 2,3,6,22,23-Pentahydroxy-2,6,11,15,19,23-hexamethyl-tetracosa-7,10,14,18-tetraene (**3**) (600 MHz in $CDCl_3$)· ······················21

Figure S19. ^{13}C NMR spectrum of 2,3,6,22,23-Pentahydroxy-2,6,11,15,19,23-hexamethyl-tetracosa-7,10,14,18-tetraene (**3**) (150 MHz in $CDCl_3$)··· ···················22

Figure S20. DEPT-135 NMR spectrum of 2,3,6,22,23-Pentahydroxy-2,6,11,15,19,23-hexamethyl-tetracosa-7,10,14,18-tetraene (**3**)··············· ······························23

Figure S21. ^{1}H-^{1}H COSY NMR spectrum of 2,3,6,22,23-Pentahydroxy-2,6,11,15,19,23-hexamethyl-tetracosa-7,10,14,18-tetraene (**3**)······ ·······························24
Figure S22. HSQC NMR spectrum of 2,3,6,22,23-Pentahydroxy-2,6,11,15,19,23-hexamethyl-tetracosa-7,10,14,18-tetraene (**3)**··················· ··························25
Figure S23. HMBC NMR spectrum of 2,3,6,22,23-Pentahydroxy-2,6,11,15,19,23-hexamethyl-tetracosa-7,10,14,18-tetraene (**3)**················· ····························26
Figure S24. HRESI-MS spectrum of 2,3,6,22,23-Pentahydroxy-2,6,11,15,19,23-hexamethyl-tetracosa-7,10,14,18-tetraene (**3)**····················· ·························27
Figure S25. ^{1}H NMR spectrum of 3,22-bis-(*S*)-MTPA ester of **3** (**3a**) ···28
Figure S26. ^{1}H NMR spectrum of 3,22-bis-(*R*)-MTPA ester of **3** (**3b**) ···29
Figure S27. ^{1}H NMR spectrum of 2,3,6,22,23-pentahydroxy-2,10,15,19,23-hexamethyl-7-methylenetetracosa-10,14,18-triene (**4**) ···30
Figure S28. ^{13}C NMR spectrum of 2,3,6,22,23-pentahydroxy-2,10,15,19,23-hexamethyl-7-methylenetetracosa-10,14,18-triene (**4**) ···31
Figure S29. DEPT-135 NMR spectrum of 2,3,6,22,23-pentahydroxy-2,10,15,19,23-hexamethyl-7-methylenetetracosa-10,14,18-triene (**4**) ·····························32
Figure S30. ^{1}H-^{1}H COSY NMR spectrum of 2,3,6,22,23-pentahydroxy-2,10,15,19,23-hexamethyl-7-methylenetetracosa-10,14,18-triene (**4**) ·························33
Figure S31. HMQC NMR spectrum of 2,3,6,22,23-pentahydroxy-2,10,15,19,23-hexamethyl-7-methylenetetracosa-10,14,18-triene (**4**) ·································34
Figure S32. HMBC NMR spectrum of 2,3,6,22,23-pentahydroxy-2,10,15,19,23-hexamethyl-7-methylenetetracosa-10,14,18-triene (**4**) ··································35
Figure S33. HRESI-MS spectrum of 2,3,6,22,23-pentahydroxy-2,10,15,19,23-hexamethyl-7-methylenetetracosa-10,14,18-triene (**4**) ·····································36
Figure S34. Inhibitory effects of compounds **2**-**4** on IL-6/STAT3 activation (a) and cell viability (b) in Hep3B cells) ···37
Figure S35. HPLC chromatogram of **1**, **2**, and **4** compounds of *A. katsumadai* ethanol-soluble extract ··38
Table S1. Inhibitory effects of EtOH extract, $CHCl_3$ and H_2O layer of *A. katsumadai* on IL-6-induced STAT3 activation ···39

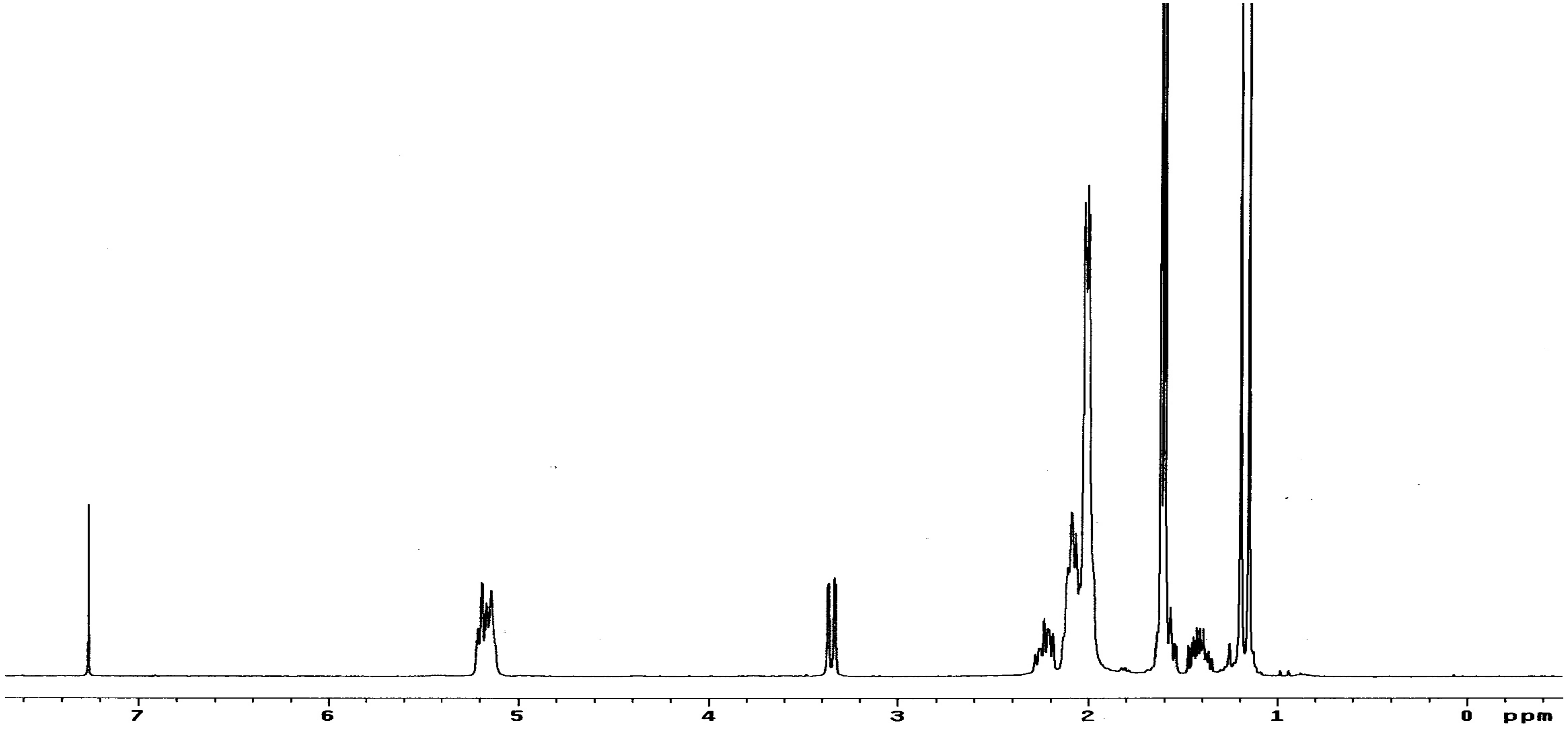

Figure S1. ^{1}H NMR spectrum of 2,3,22,23-tertrahydroxy-2,6,10,15,19,23-hexamethyl-tetracosa-6,10,14,18-tetraene (**1**) (300 MHz in $CDCl_3$).

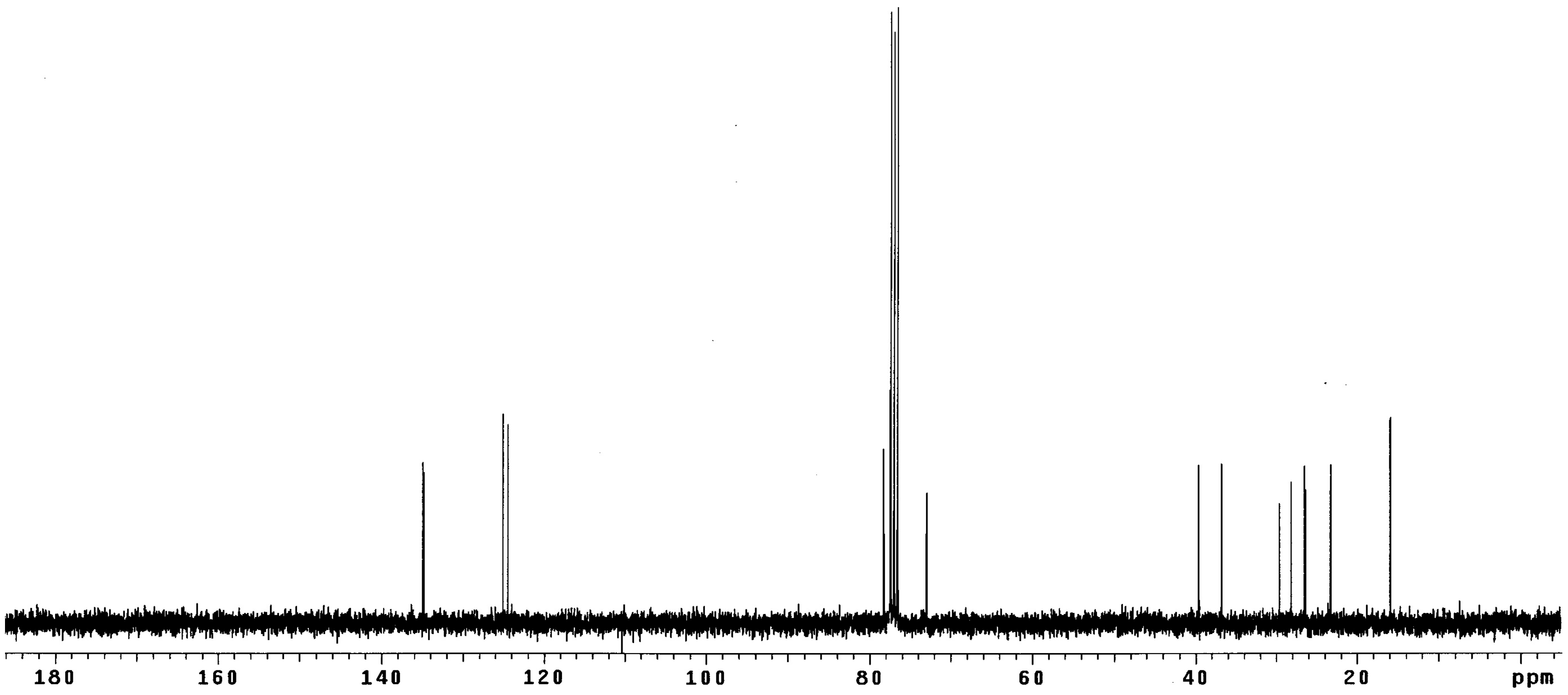

Figure S2. ^{13}C NMR spectrum of 2,3,22,23-tertrahydroxy-2,6,10,15,19,23-hexamethyl-tetracosa-6,10,14,18-tetraene (**1**) (75 MHz in $CDCl_3$).

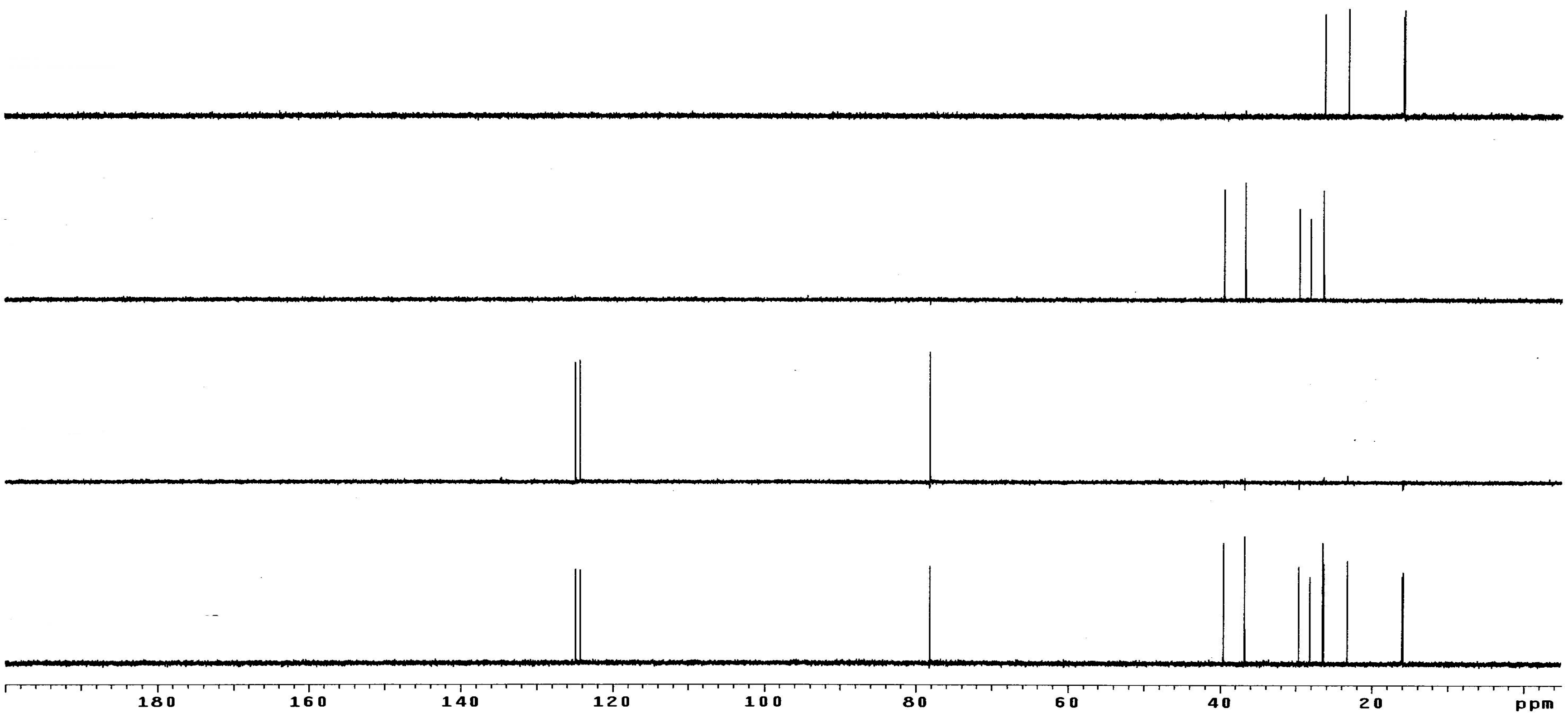

Figure S3. DEPT NMR spectrum of 2,3,22,23-tertrahydroxy-2,6,10,15,19,23-hexamethyl-tetracosa-6,10,14,18-tetraene (**1**) (75 MHz in $CDCl_3$).

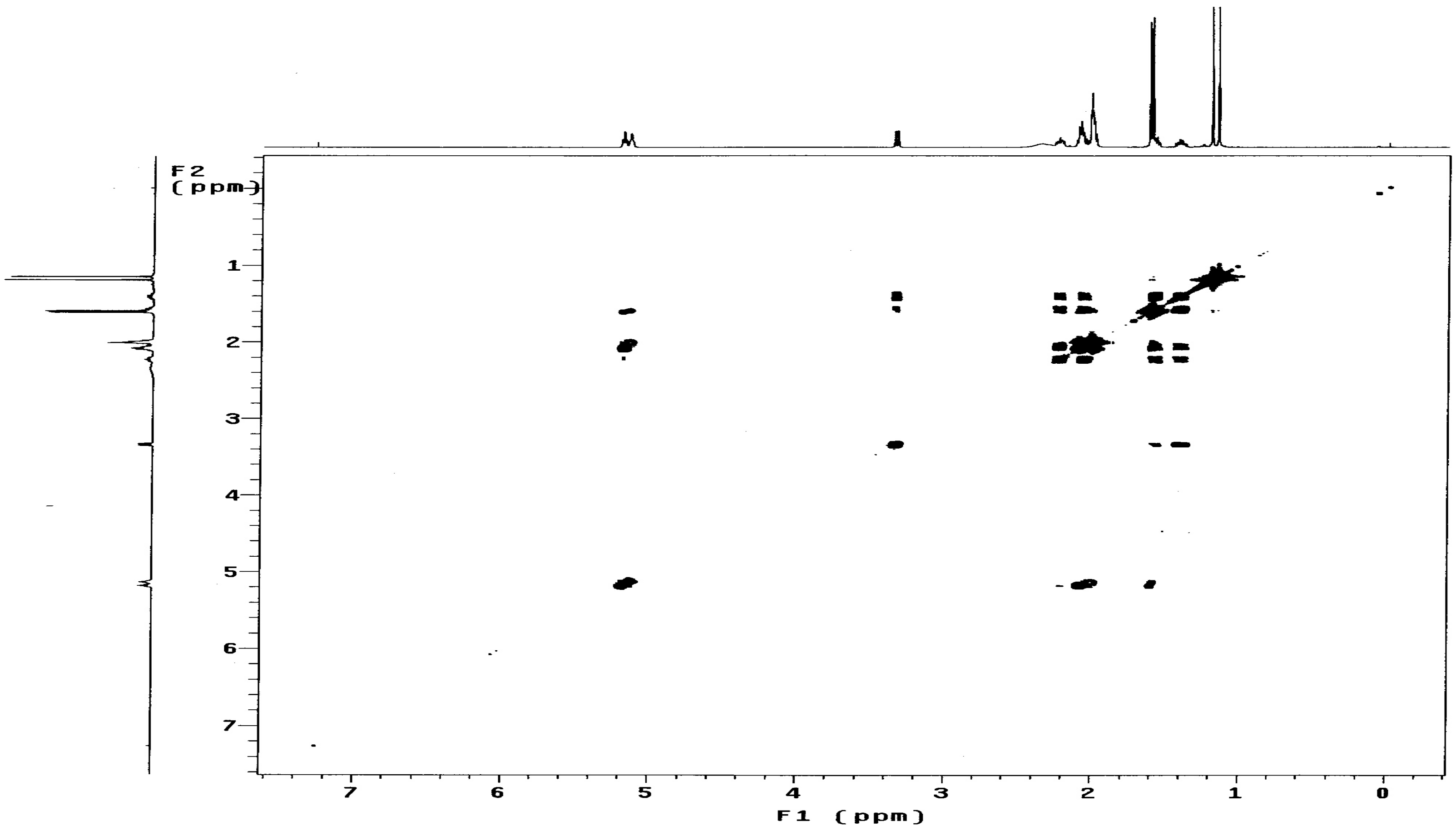

Figure S4. ^{1}H-^{1}H COSY NMR spectrum of 2,3,22,23-tertrahydroxy-2,6,10,15,19,23-hexamethyl-tetracosa-6,10,14,18-tetraene (**1**).

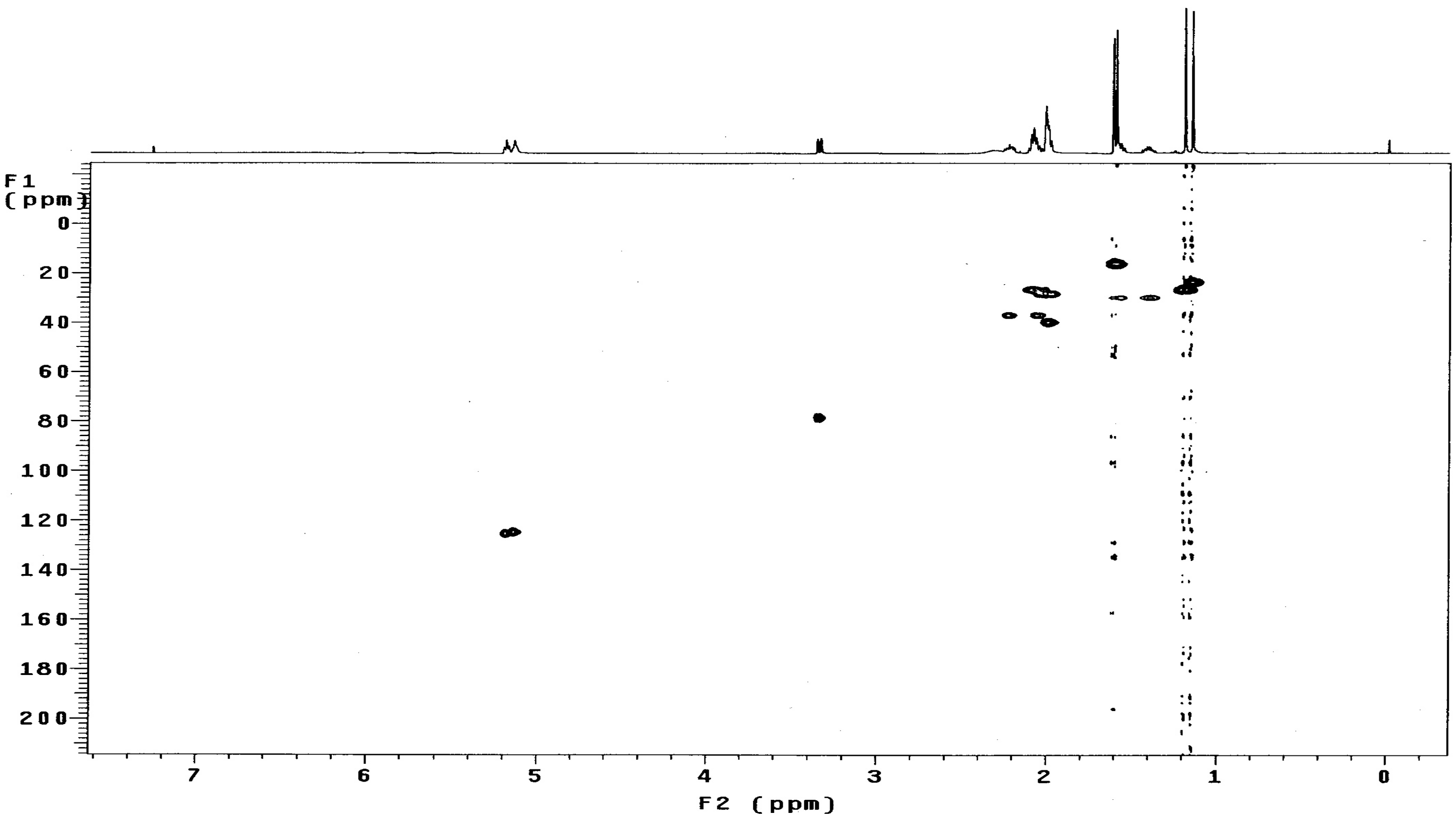

Figure S5. HMQC NMR spectrum of 2,3,22,23-tertrahydroxy-2,6,10,15,19,23-hexamethyl-tetracosa-6,10,14,18-tetraene (**1**).

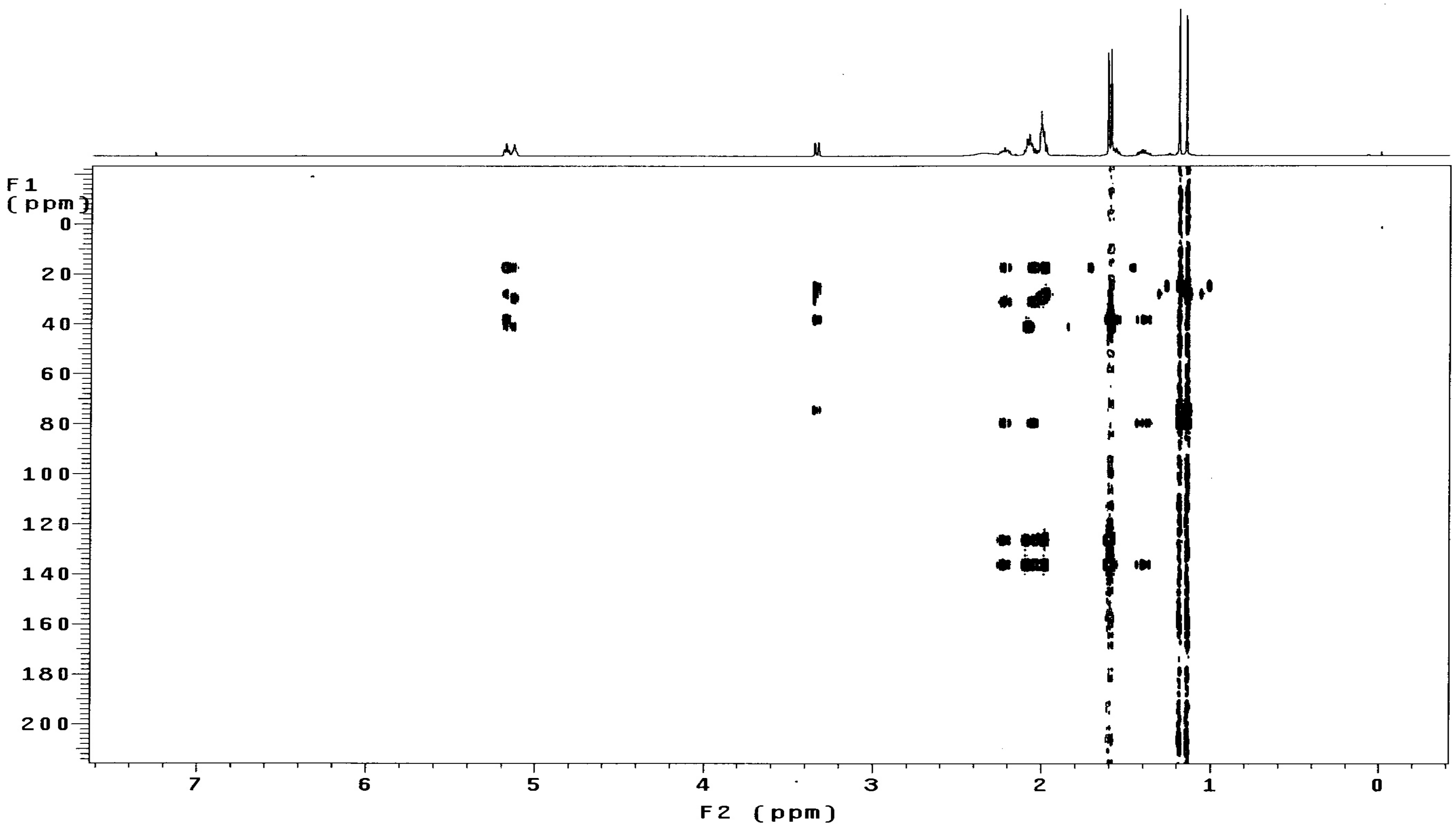

Figure S6. HMBC NMR spectrum of 2,3,22,23-tertrahydroxy-2,6,10,15,19,23-hexamethyl-tetracosa-6,10,14,18-tetraene (**1**).

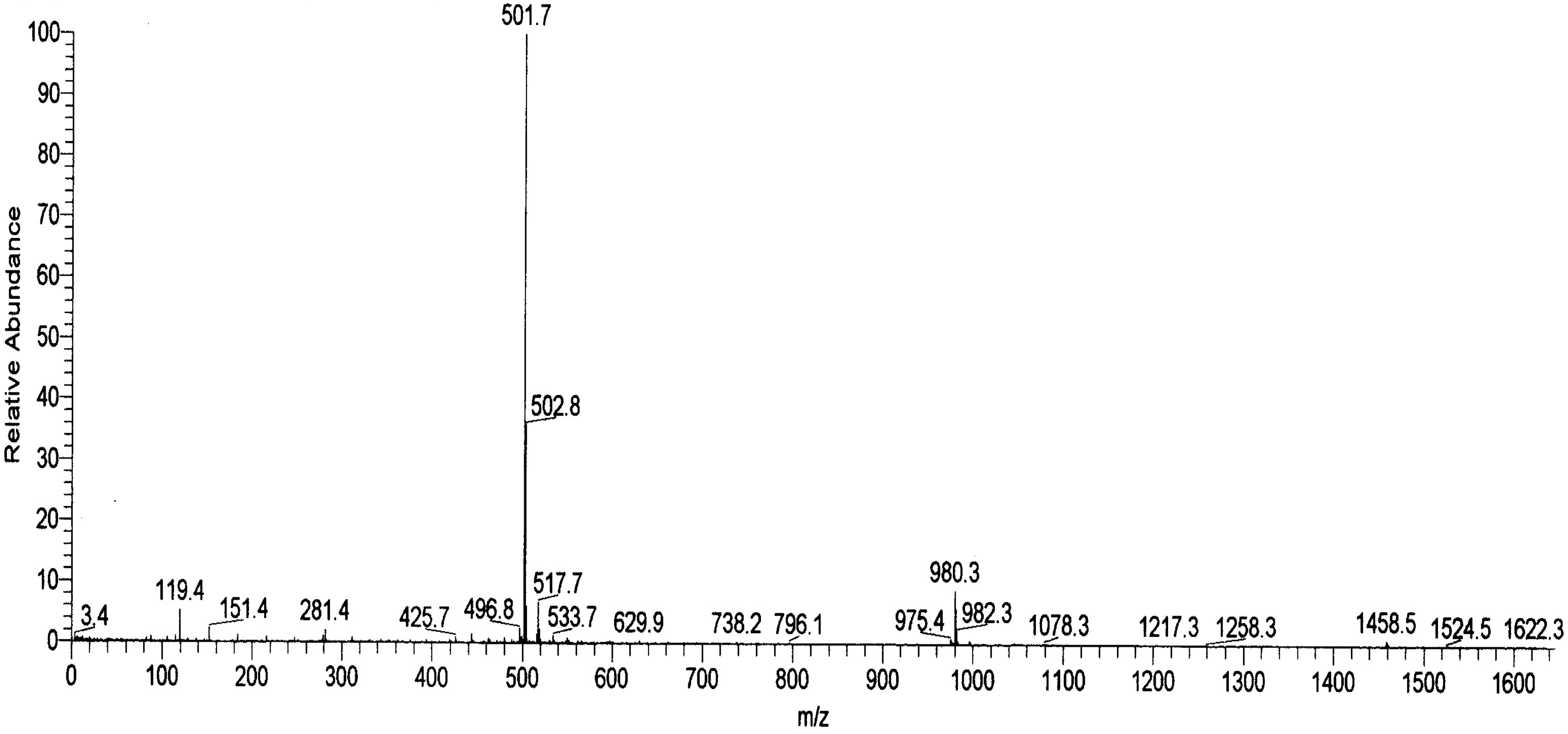

Figure S7. LRESIMS spectrum of 2,3,22,23-tertrahydroxy-2,6,10,15,19,23-hexamethyl-tetracosa-6,10,14,18-tetraene (**1**).

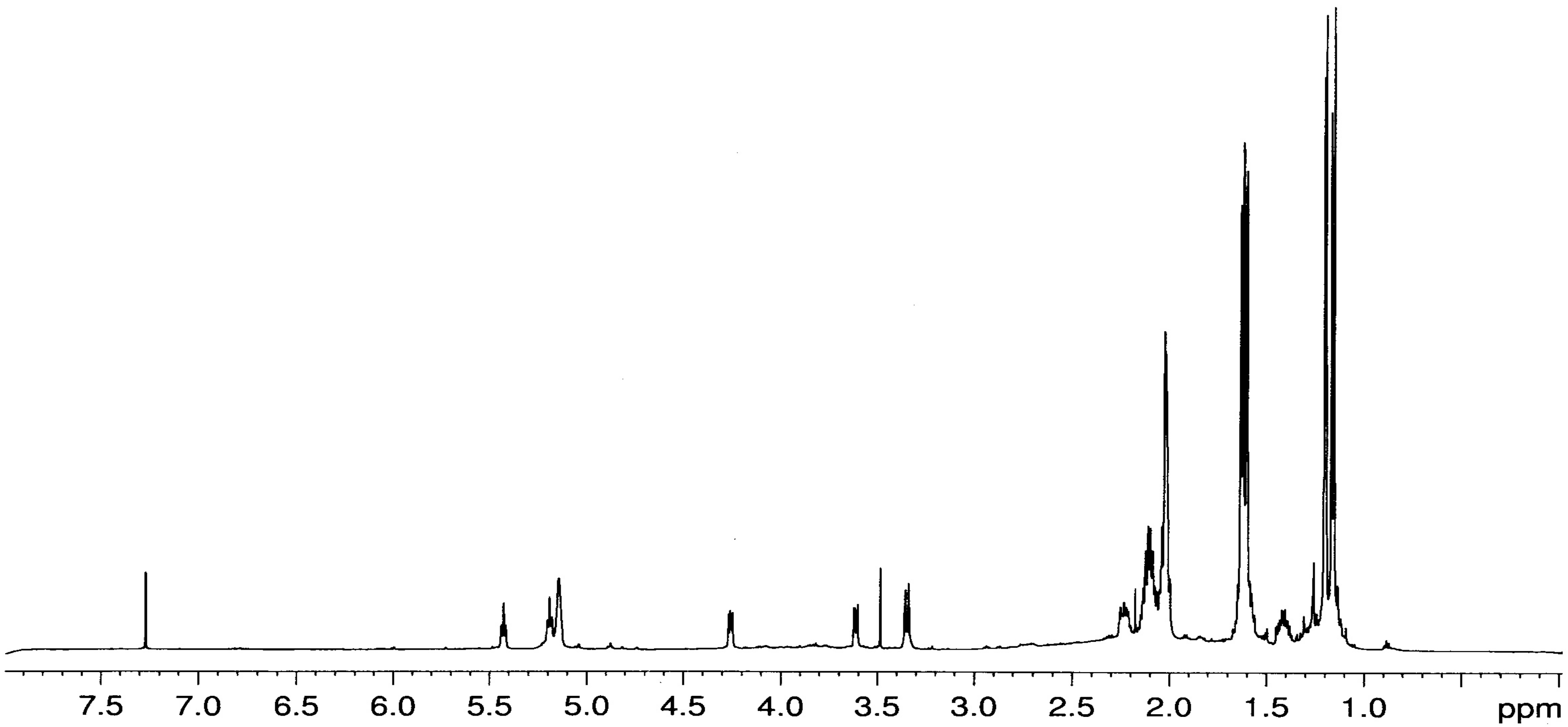

Figure S8. ^{1}H NMR spectrum of 2,3,5,22,23-pentahydroxy-2,6,10,15,19,23-hexamethyl-tetracosa-6,10,14,18-tetraene (**2**) (500 MHz in $CDCl_3$).

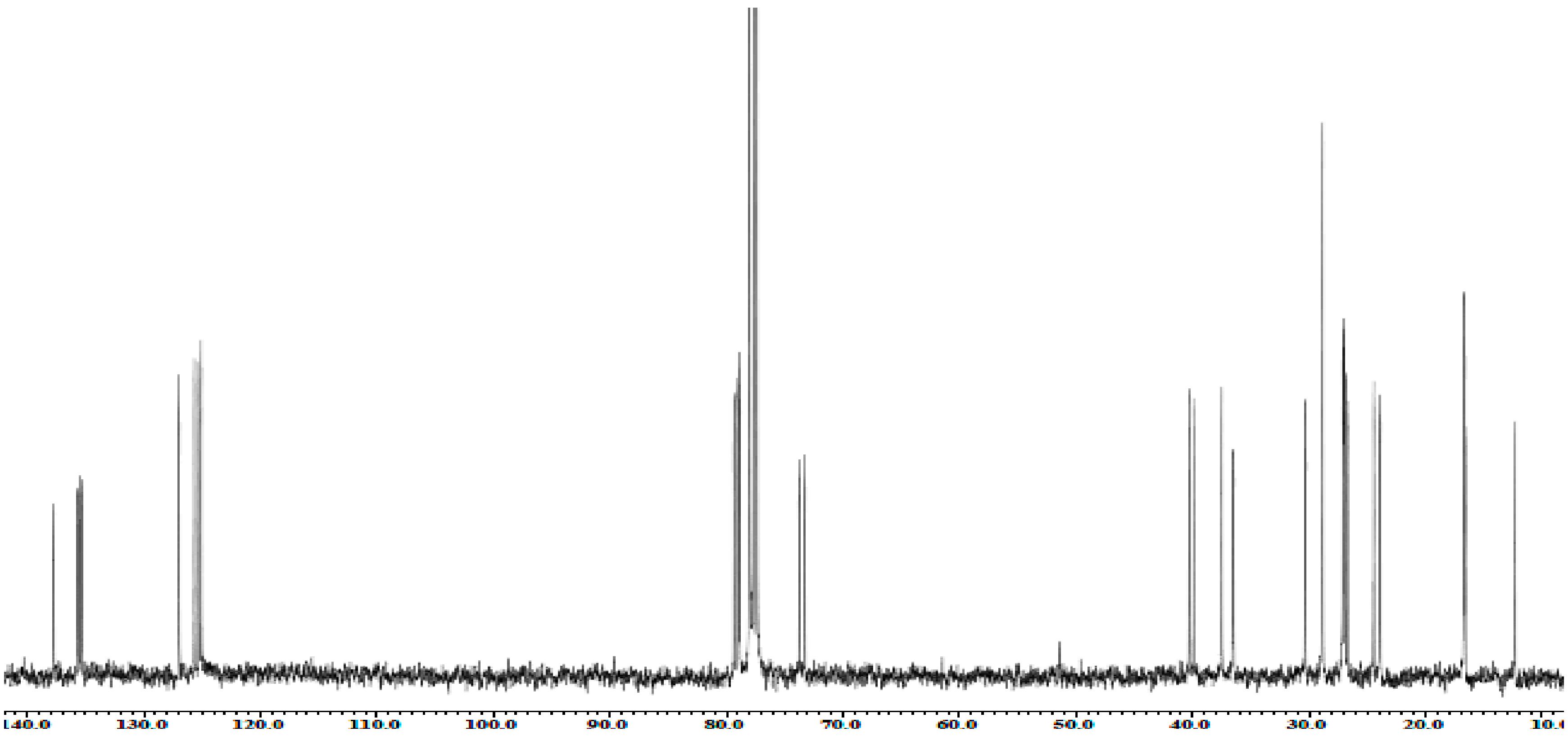

Figure S9. ^{13}C NMR spectrum of 2,3,5,22,23-pentahydroxy-2,6,10,15,19,23-hexamethyl-tetracosa-6,10,14,18-tetraene (**2**) (125 MHz in $CDCl_3$).

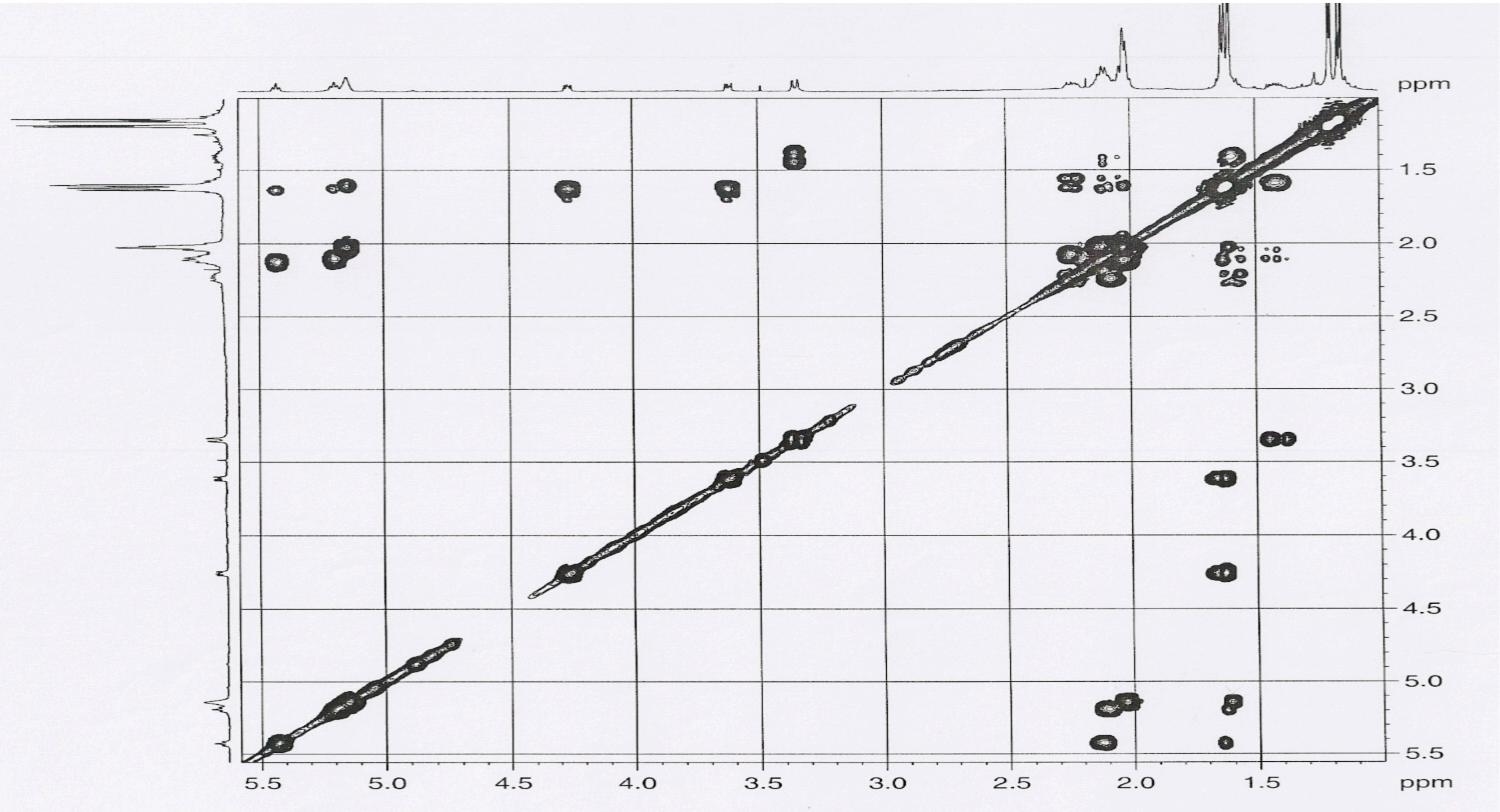

Figure S10. ^{1}H-^{1}H COSY NMR spectrum of 2,3,5,22,23-pentahydroxy-2,6,10,15,19,23-hexamethyl-tetracosa-6,10,14,18-tetraene (**2**).

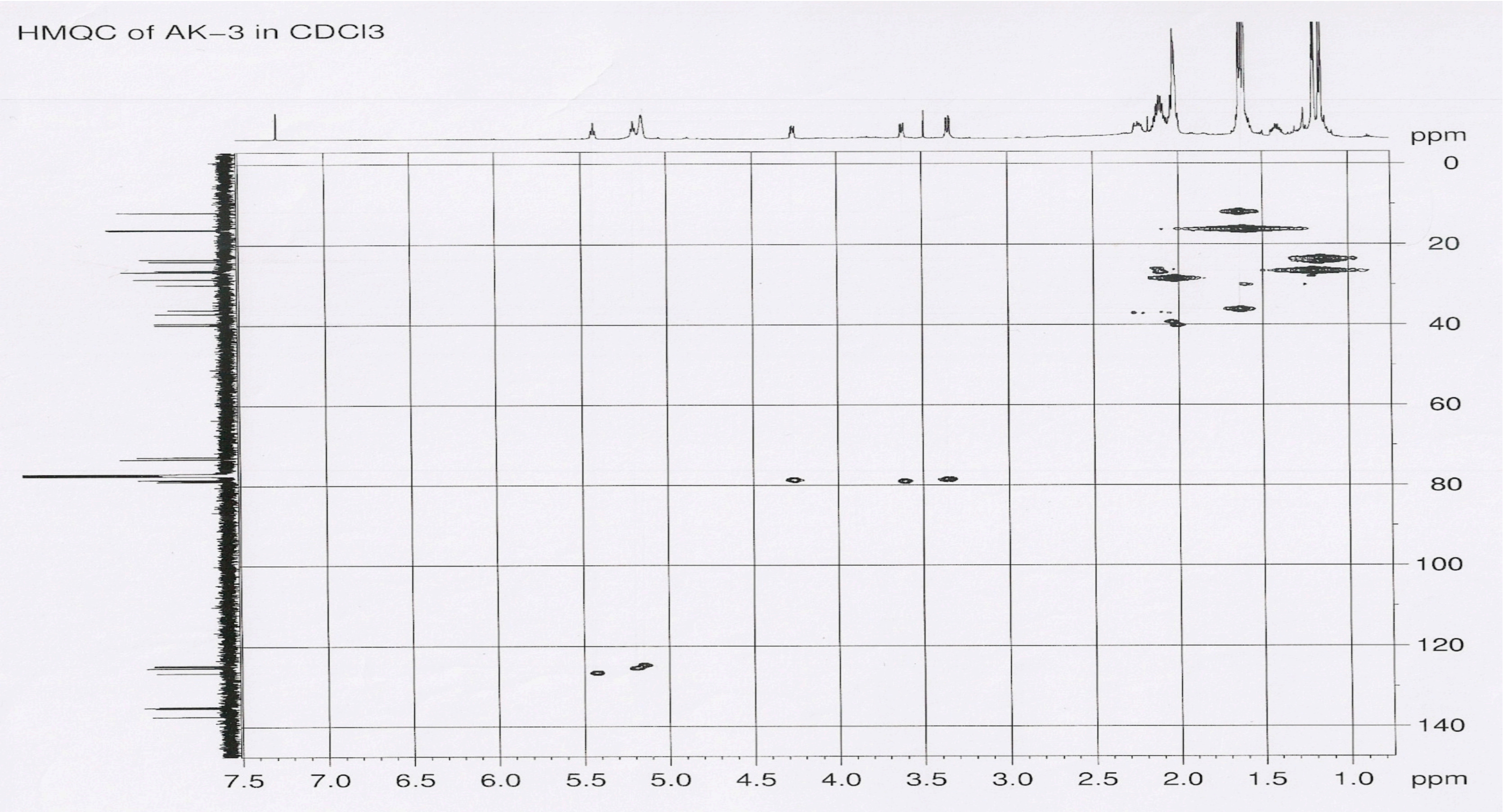

Figure S11. HMQC NMR spectrum of 2,3,5,22,23-pentahydroxy-2,6,10,15,19,23-hexamethyl-tetracosa-6,10,14,18-tetraene (**2**).

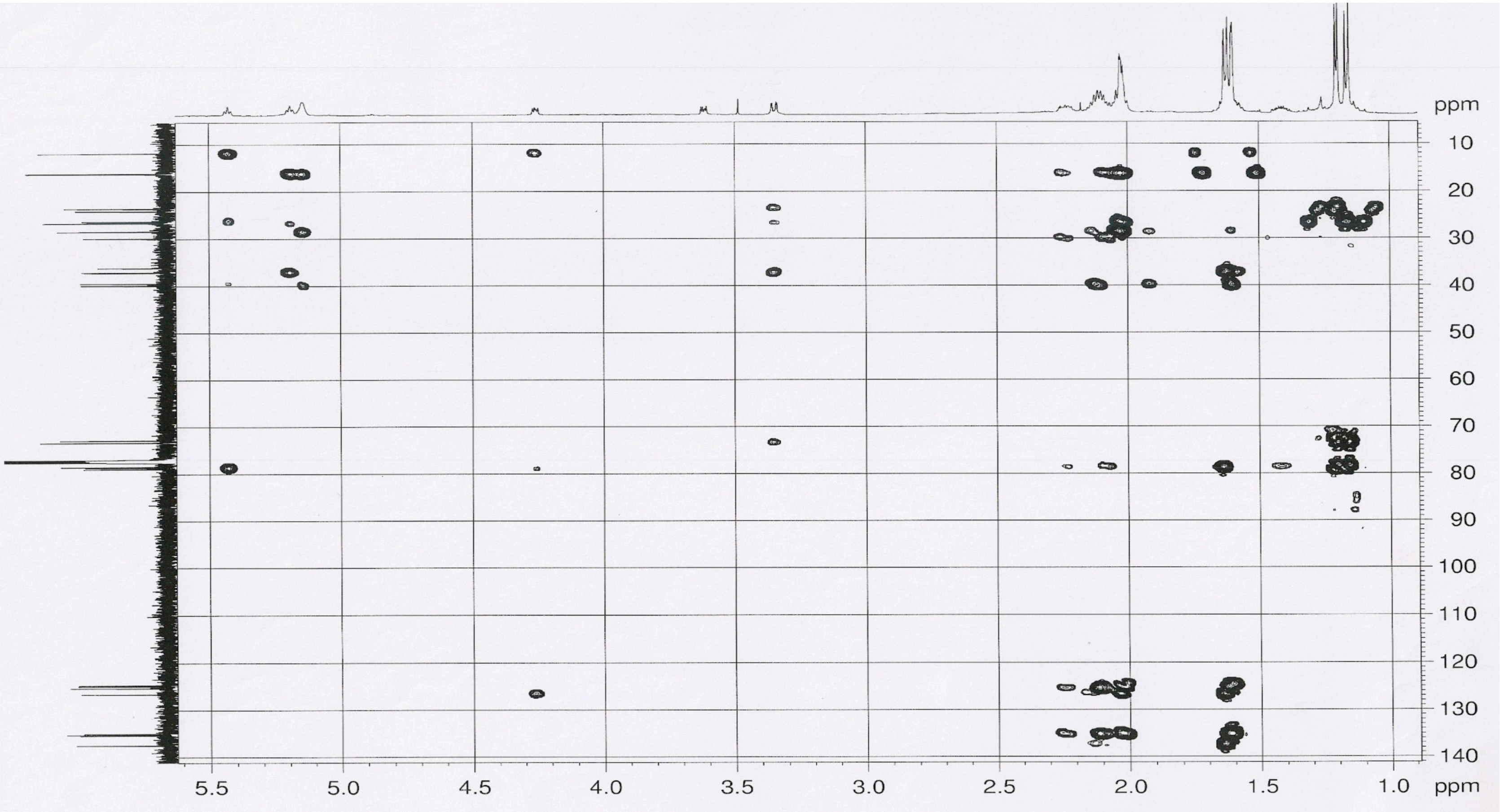

Figure S12. HMBC NMR spectrum of 2,3,5,22,23-pentahydroxy-2,6,10,15,19,23-hexamethyl-tetracosa-6,10,14,18-tetraene (**2**).

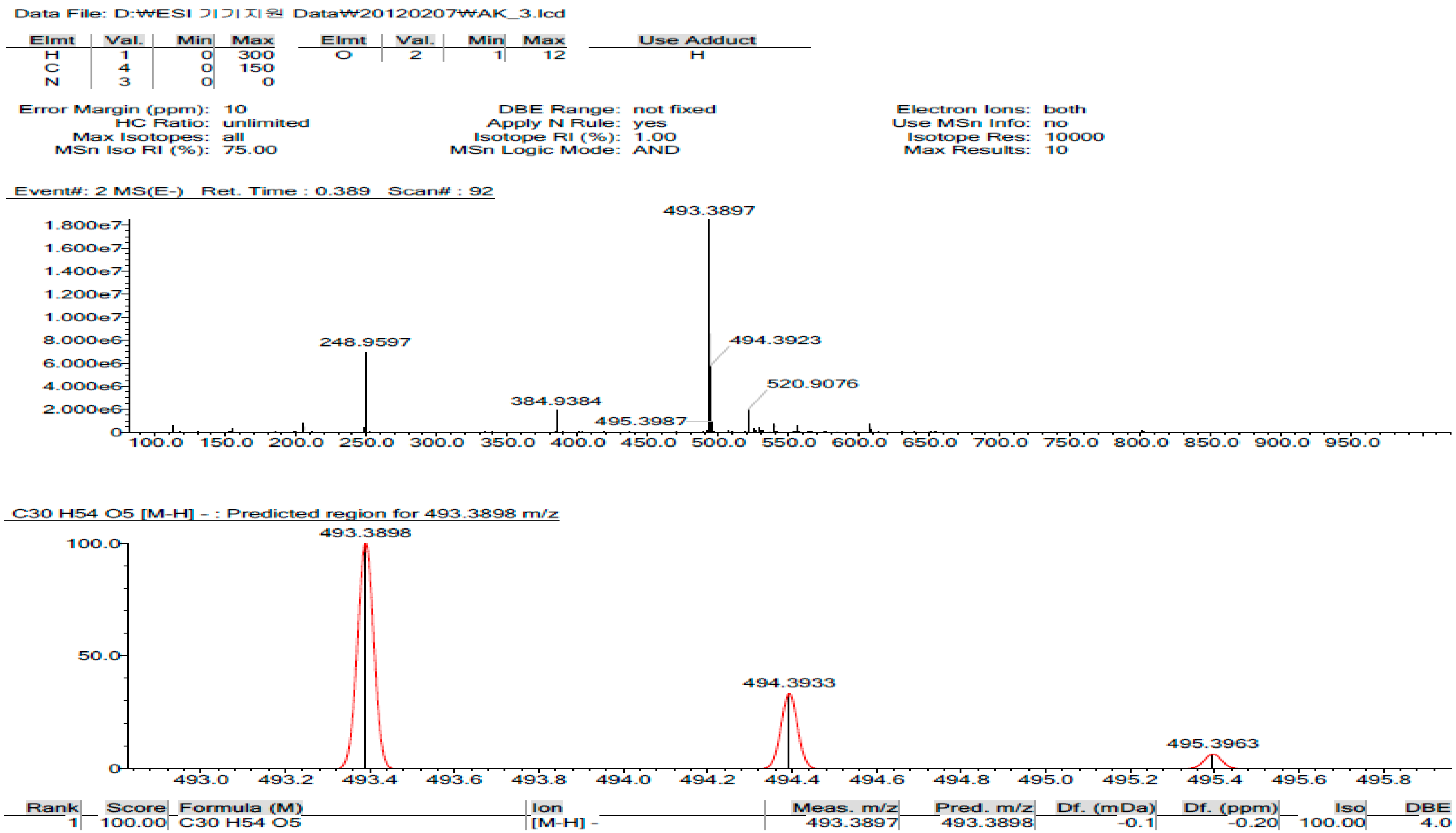

Figure S13. HRESIMS spectrum of 2,3,5,22,23-pentahydroxy-2,6,10,15,19,23-hexamethyl-tetracosa-6,10,14,18-tetraene (**2**).

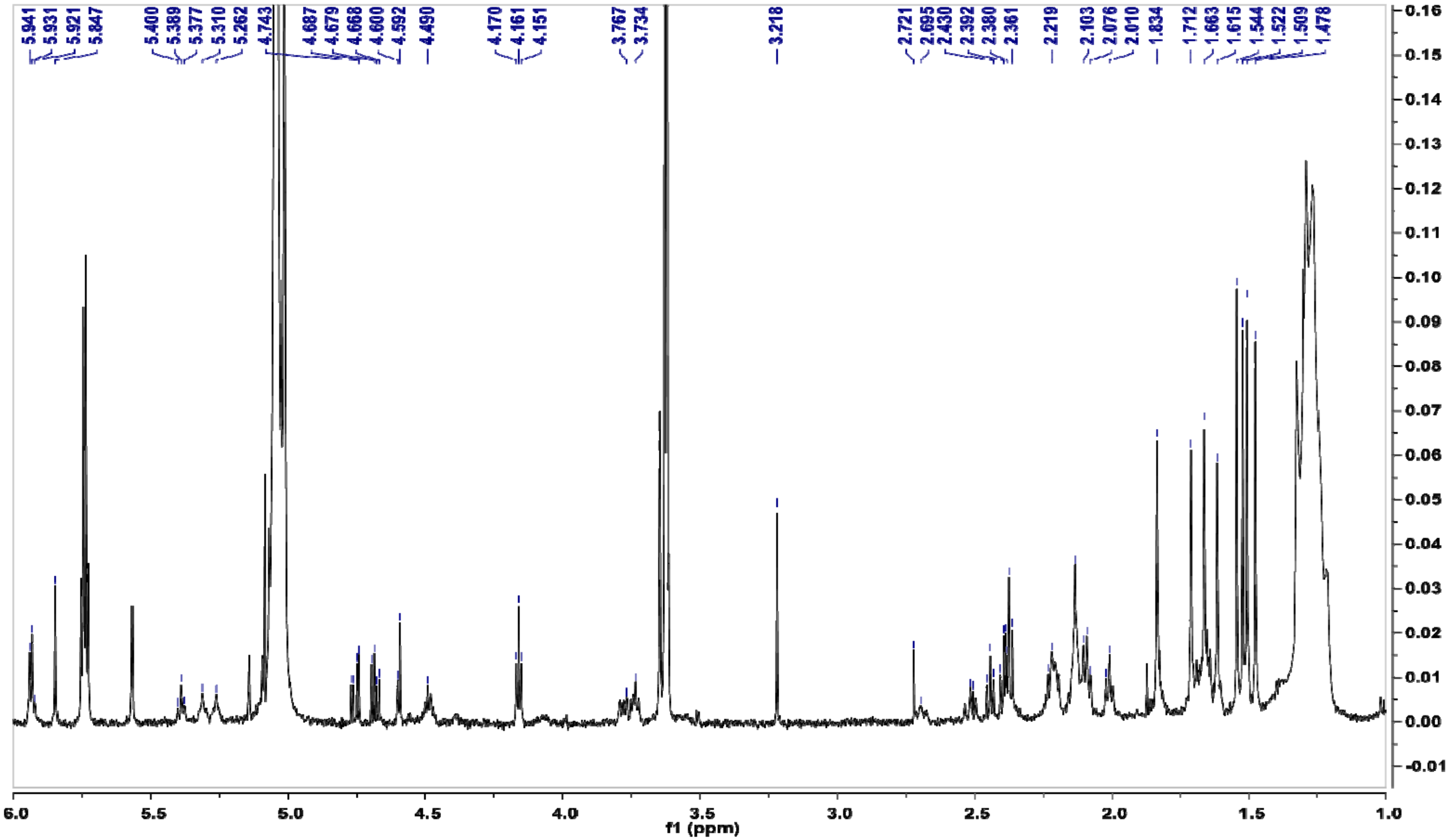

Figure S14. ^{1}H NMR spectrum of 5-mono-(*S*)-MTPA ester of **2** (**2a**) (600 MHz in pyridine-d_5).

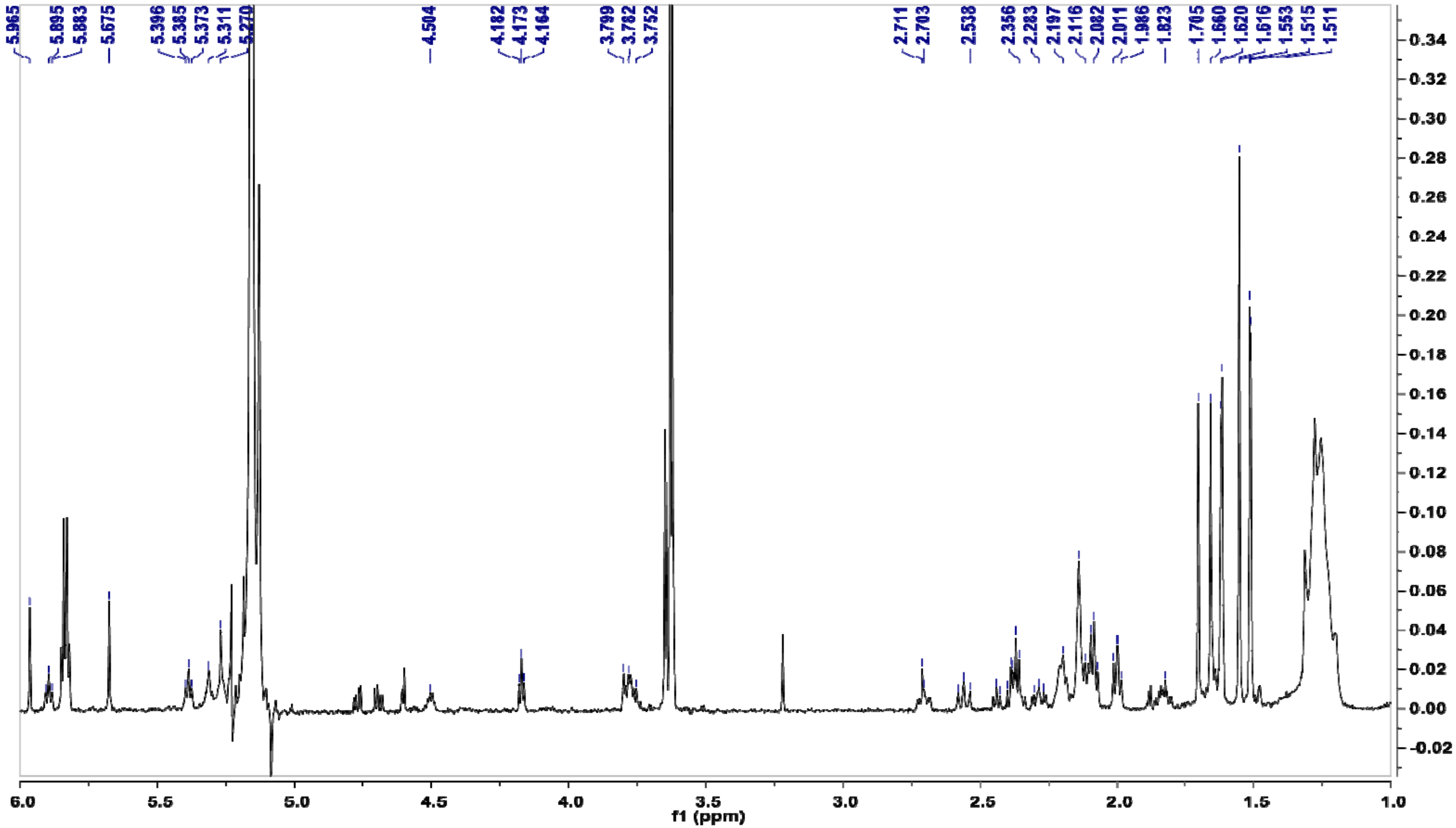

Figure S15. ^{1}H NMR spectrum of 5-mono-(*R*)-MTPA ester of **2** (**2c**) (600 MHz in pyridine-d_5).

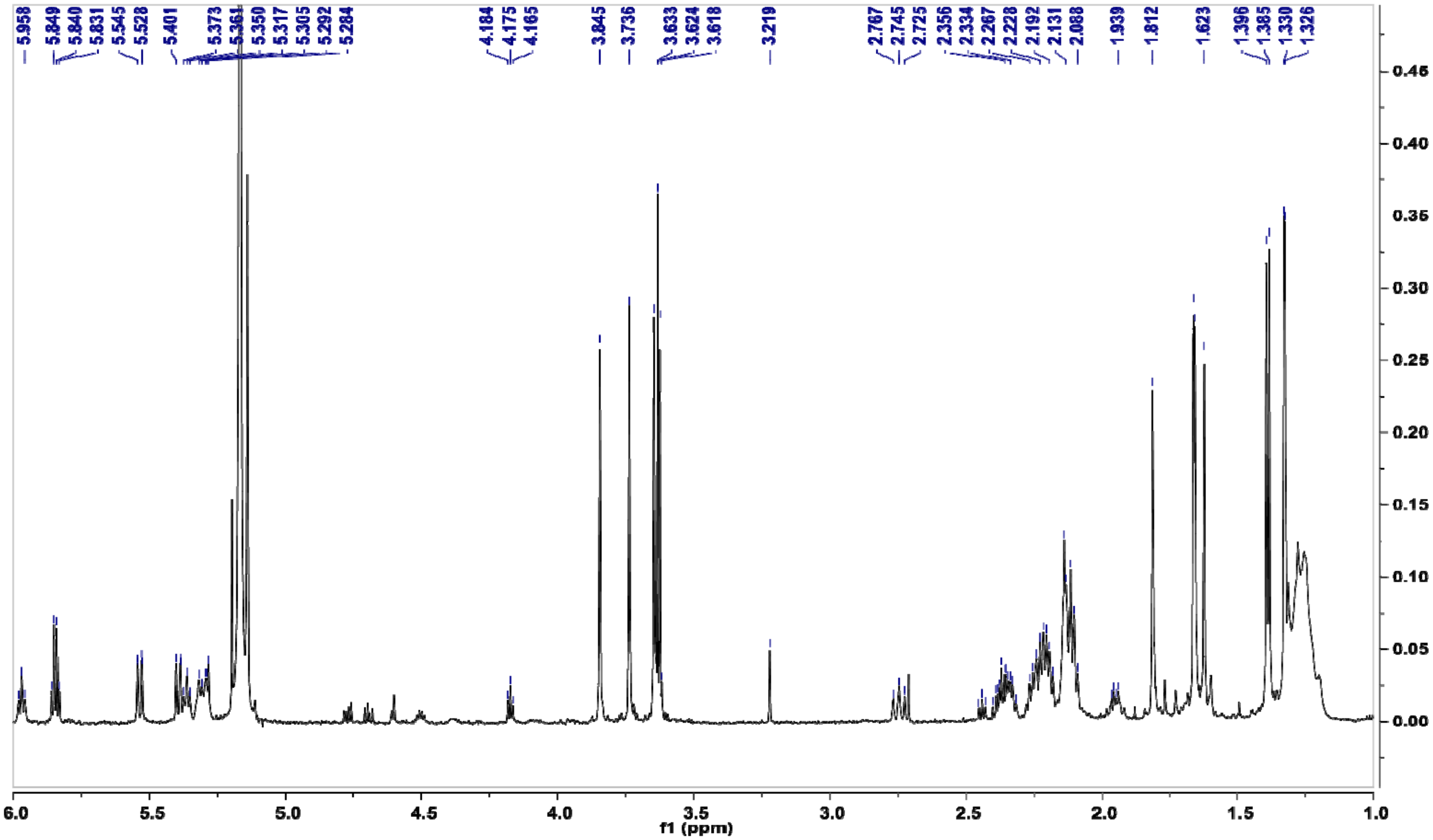

Figure S16. ^{1}H NMR spectrum of 3,5,22-tris-(*S*)-MTPA ester of **2** (**2b**) (600 MHz in pyridine-d_5).

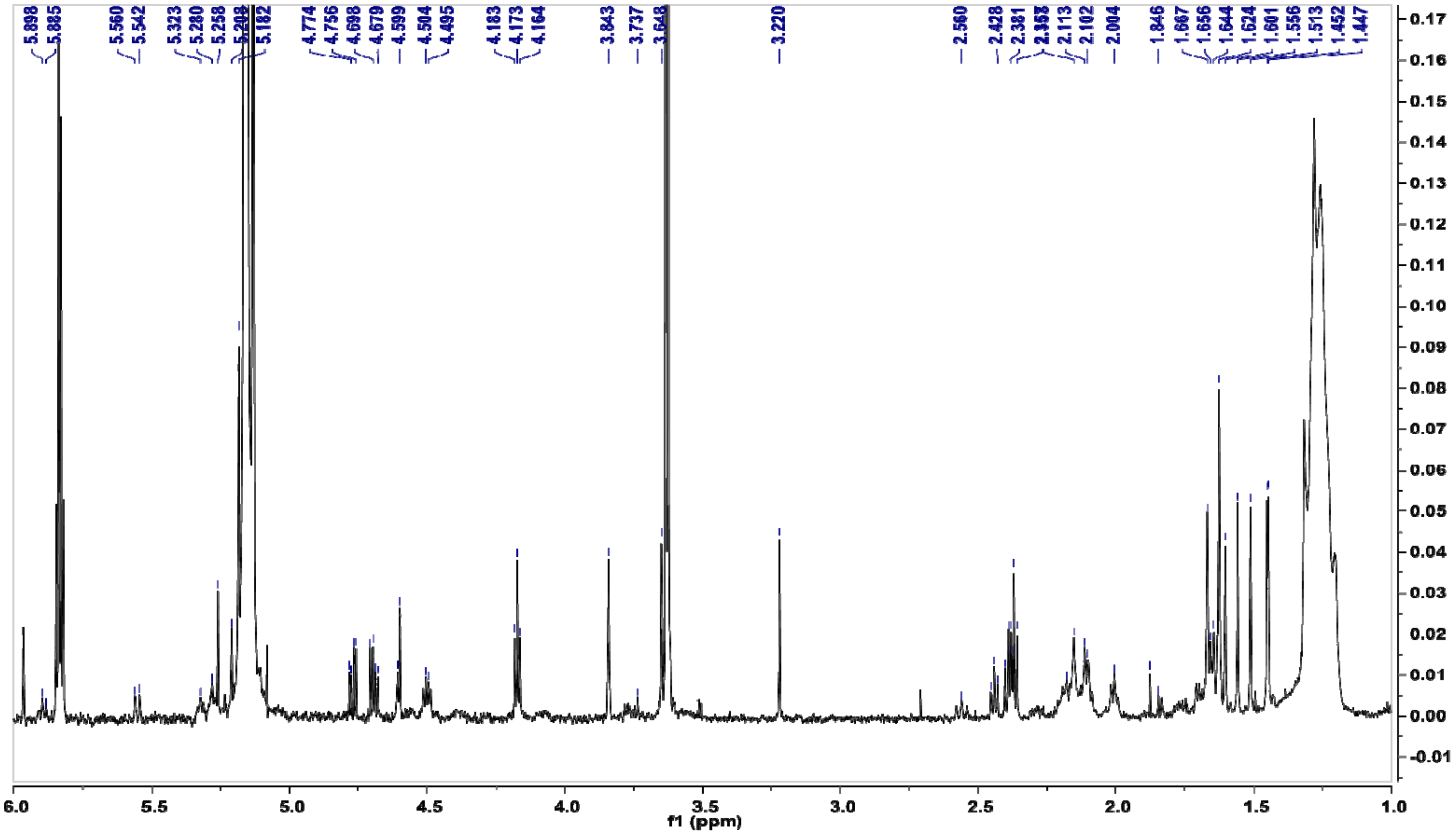

Figure S17. ^{1}H NMR spectrum of 3,5,22-tris-(*R*)-MTPA ester of **2** (**2d**) (600 MHz in pyridine-d_5).

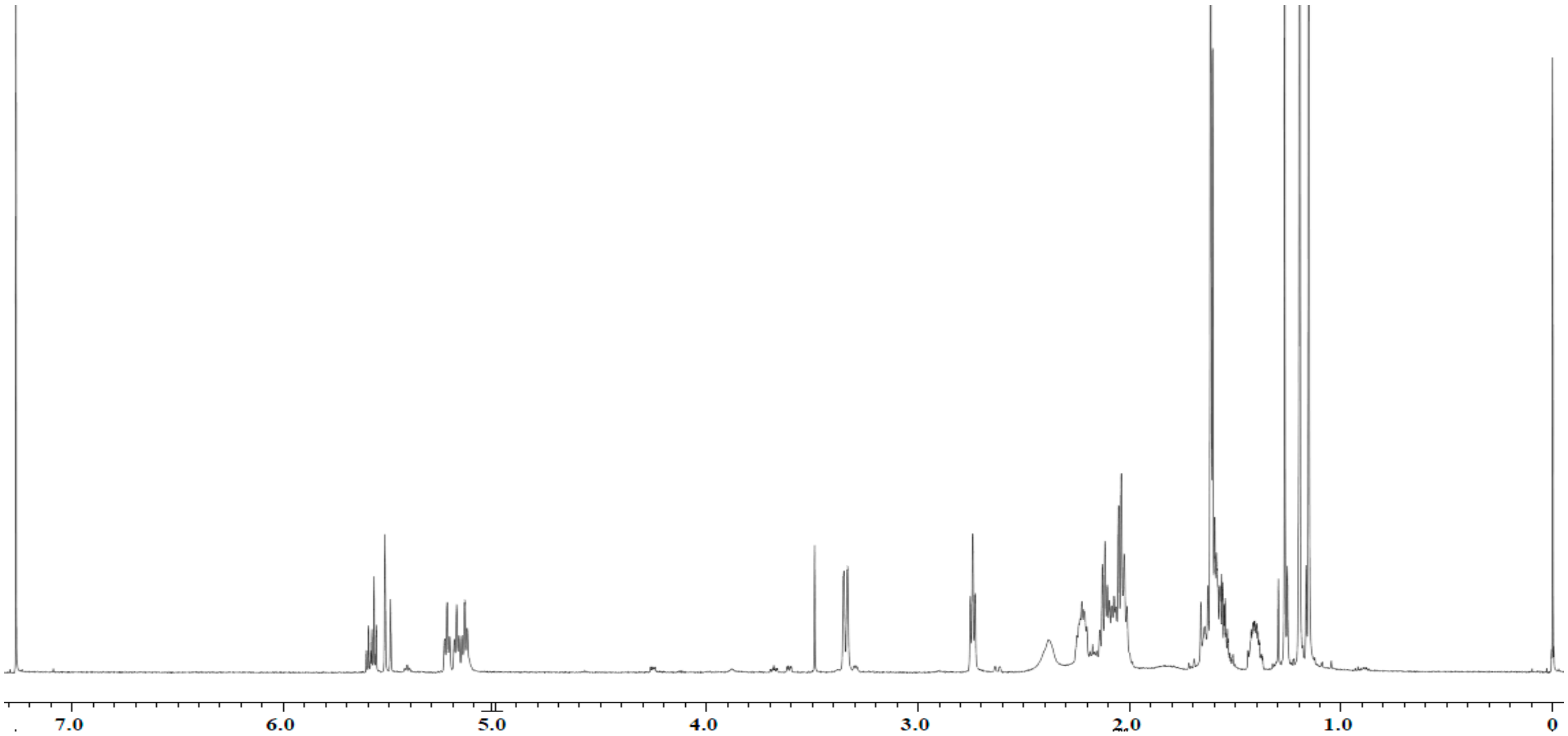

Figure S18. ^{1}H NMR spectrum of 2,3,6,22,23-Pentahydroxy-2,6,11,15,19,23-hexamethyl-tetracosa-7,10,14,18-tetraene (**3**) (600 MHz in $CDCl_3$).

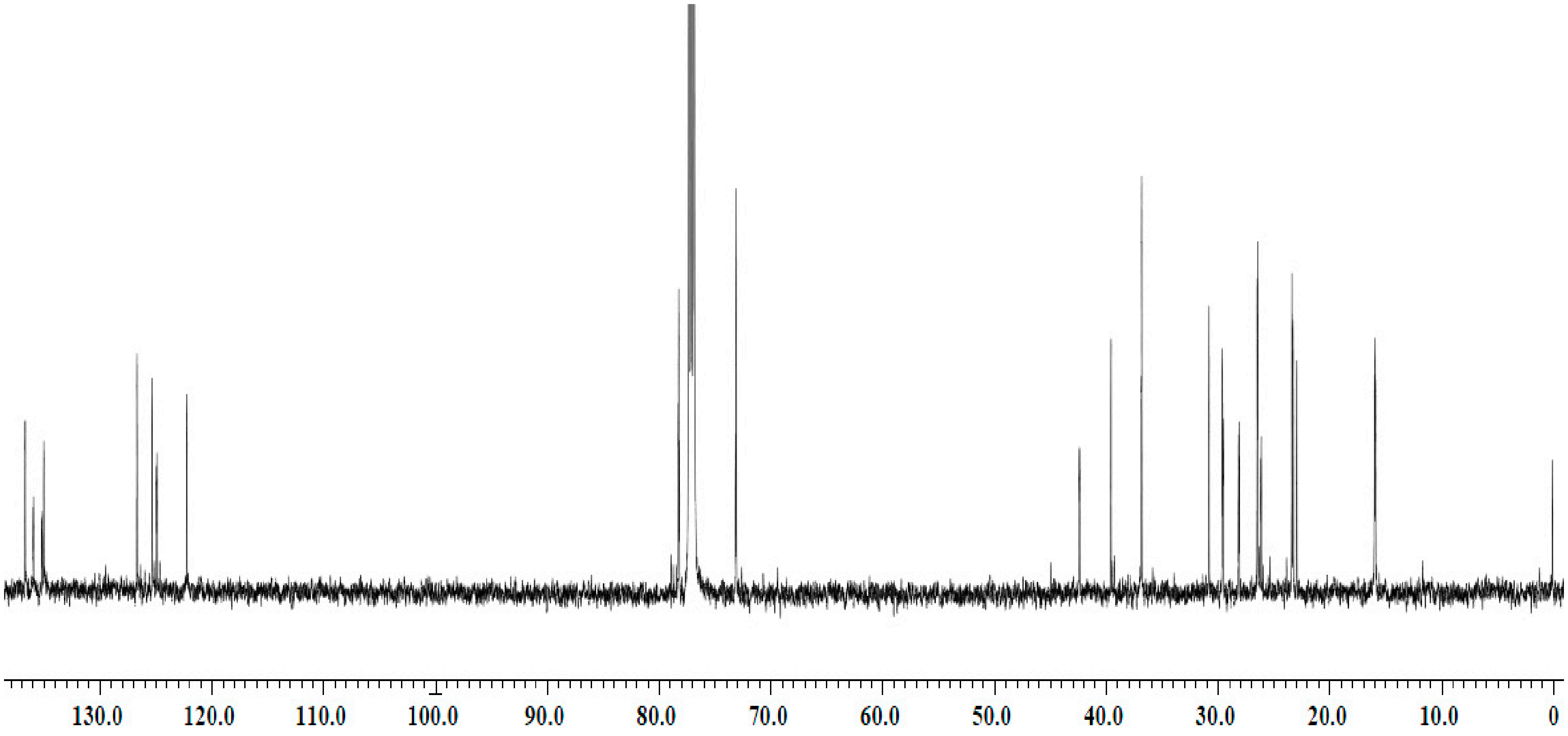

Figure S19. ^{13}C NMR spectrum of 2,3,6,22,23-Pentahydroxy-2,6,11,15,19,23-hexamethyl-tetracosa-7,10,14,18-tetraene (**3**) (150 MHz in $CDCl_3$).

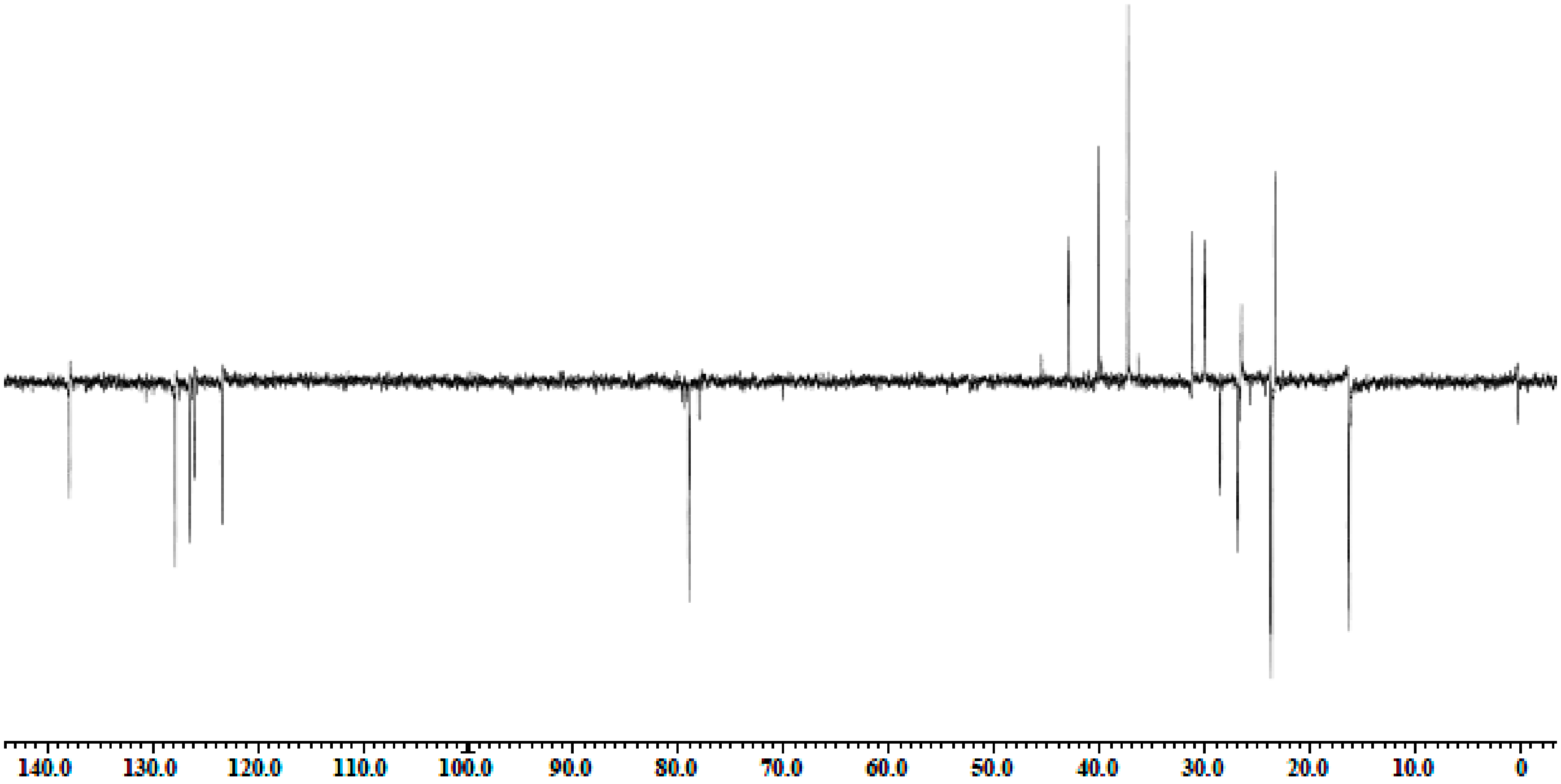

Figure S20. DEPT-135 NMR spectrum of 2,3,6,22,23-Pentahydroxy-2,6,11,15,19,23-hexamethyl-tetracosa-7,10,14,18-tetraene (**3**) (150 MHz in $CDCl_3$).

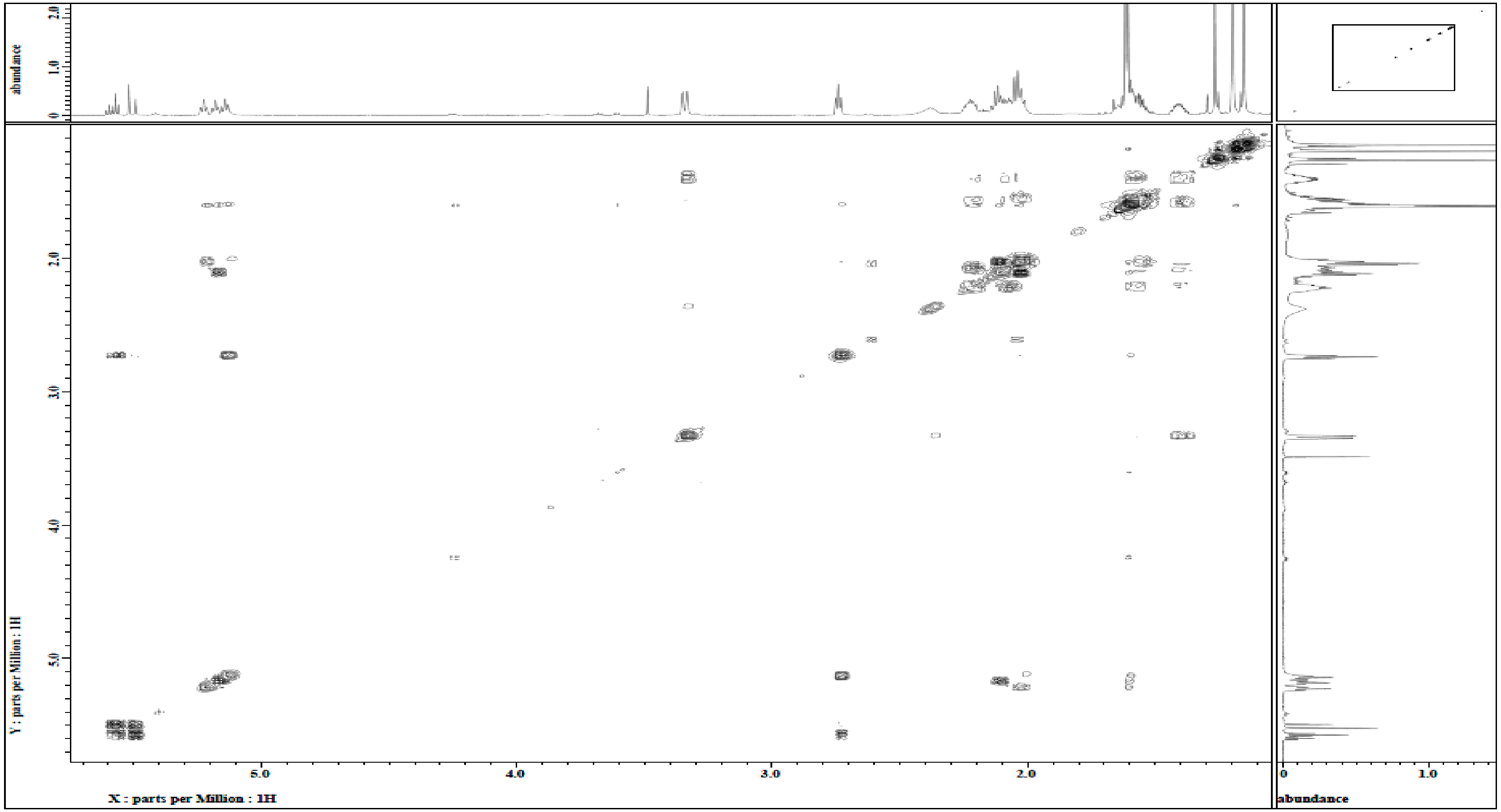

Figure S21. ^{1}H-^{1}H COSY NMR spectrum of 2,3,6,22,23-Pentahydroxy-2,6,11,15,19,23-hexamethyl-tetracosa-7,10,14,18-tetraene (**3**).

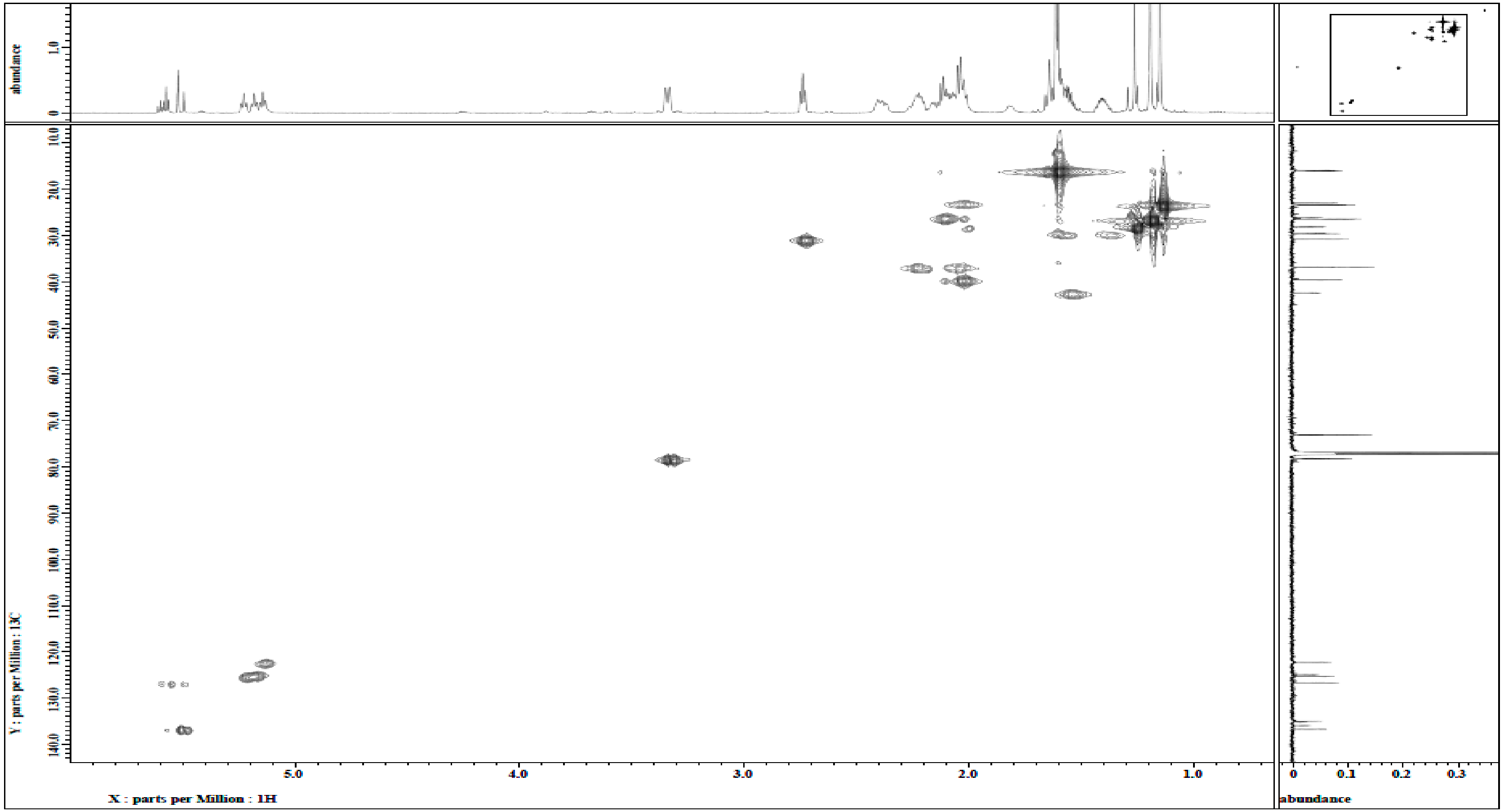

Figure S22. HSQC NMR spectrum of 2,3,6,22,23-Pentahydroxy-2,6,11,15,19,23-hexamethyl-tetracosa-7,10,14,18-tetraene (**3**).

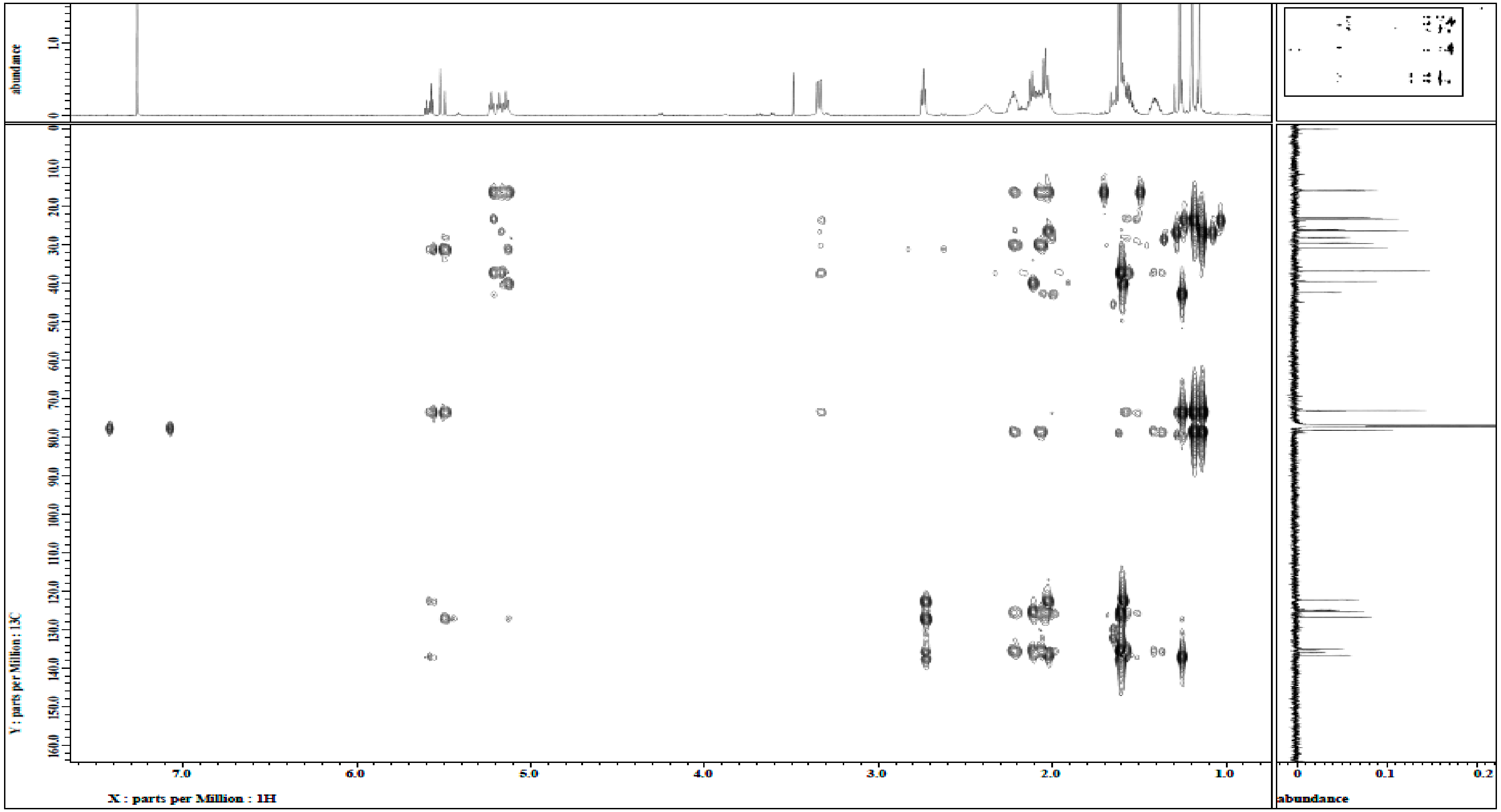

Figure S23. HMBC NMR spectrum of 2,3,6,22,23-Pentahydroxy-2,6,11,15,19,23-hexamethyl-tetracosa-7,10,14,18-tetraene (**3**).

Data File: D:₩ESI 기기지원 Data₩20111229₩CDG_2.lcd

Elmt	Val.	Min	Max	Elmt	Val.	Min	Max	Use Adduct
H	1	0	300	O	2	0	12	Na
C	4	0	150					
N	3	0	0					

Error Margin (ppm): 10
HC Ratio: unlimited
Max Isotopes: all
MSn Iso RI (%): 75.00

DBE Range: not fixed
Apply N Rule: yes
Isotope RI (%): 1.00
MSn Logic Mode: AND

Electron Ions: both
Use MSn Info: no
Isotope Res: 10000
Max Results: 10

Event#: 1 MS(E+) Ret. Time : 3.046 Scan# : 653

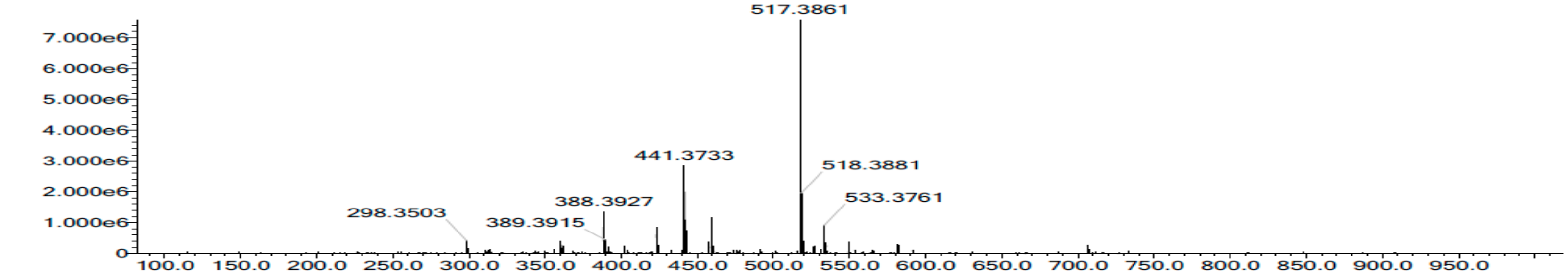

C30 H54 O5 [M+Na] + : Predicted region for 517.3863 m/z

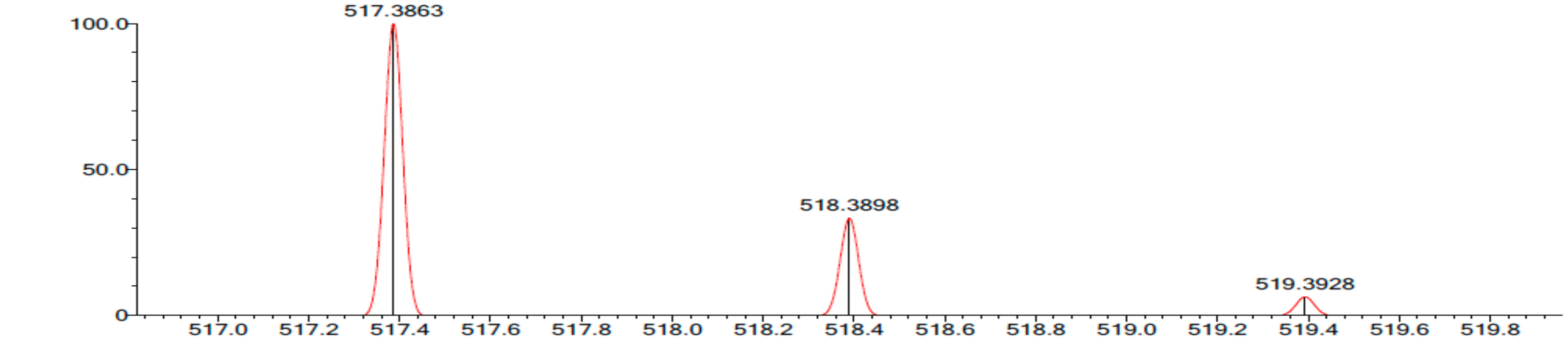

Rank	Score	Formula (M)	Ion	Meas. m/z	Pred. m/z	Df. (mDa)	Df. (ppm)	Iso	DBE
1	75.57	C30 H54 O5	[M+Na] +	517.3861	517.3863	-0.2	-0.39	75.57	4.0

Figure S24. HRESIMS spectrum of 2,3,6,22,23-Pentahydroxy-2,6,11,15,19,23-hexamethyl-tetracosa-7,10,14,18-tetraene (**3**).

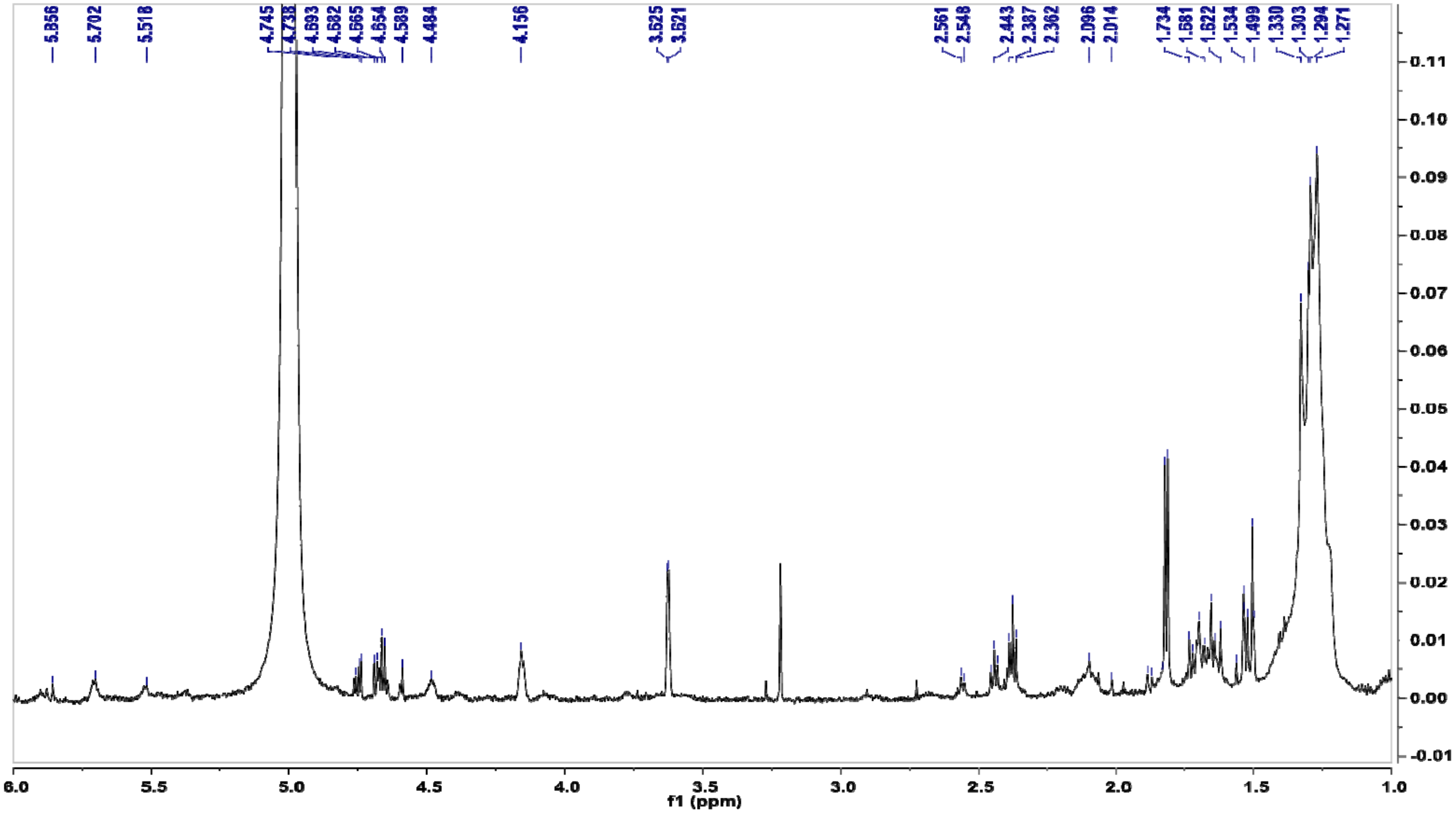

Figure S25. ^{1}H NMR spectrum of 3,22-bis-(*S*)-MTPA ester of **3** (**3a**) (600 MHz in pyridine-d_5).

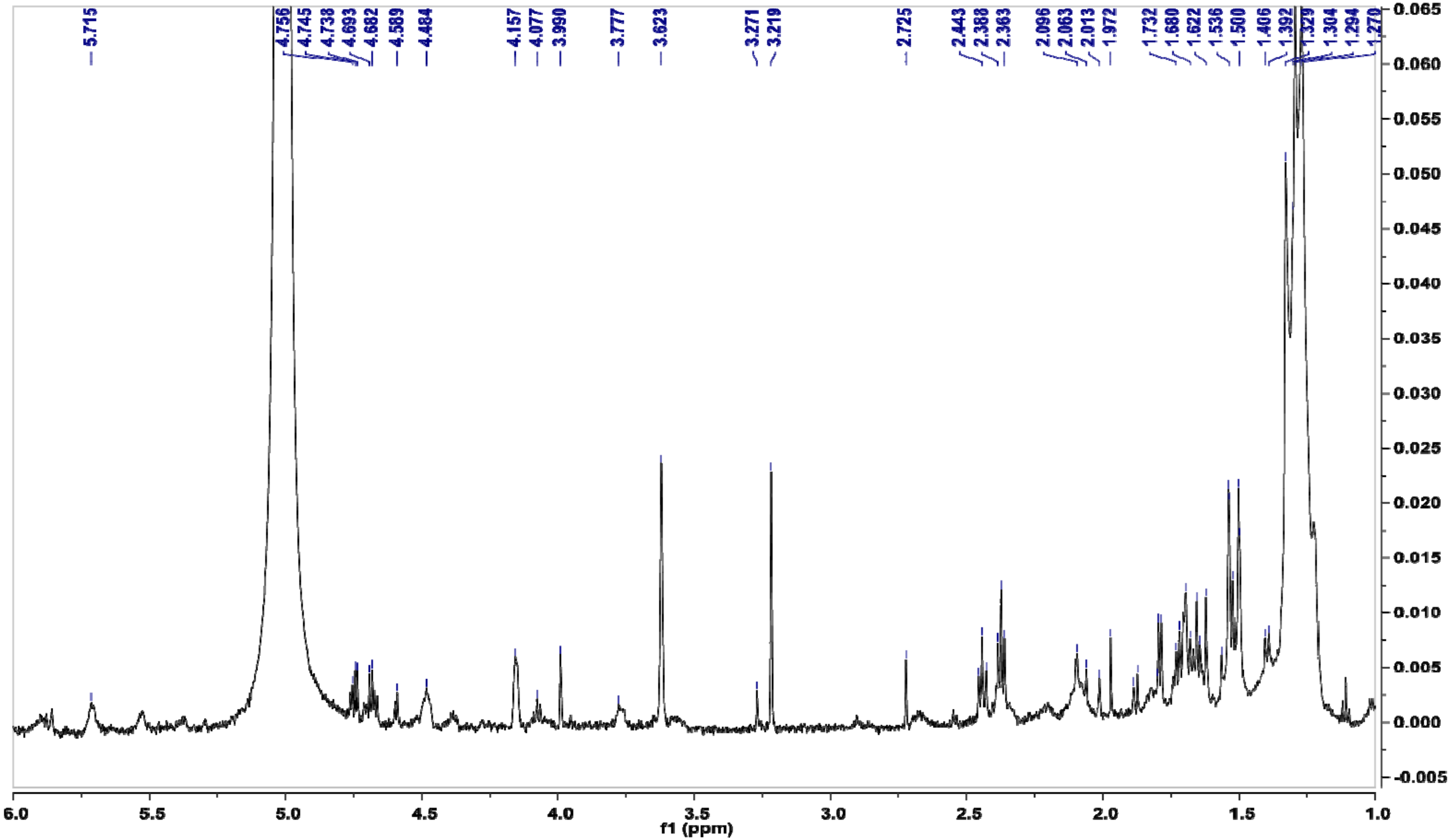

Figure S26. ^{1}H NMR spectrum of 3,22-bis-(*R*)-MTPA ester of **3** (**3b**) (600 MHz in pyridine-d_5).

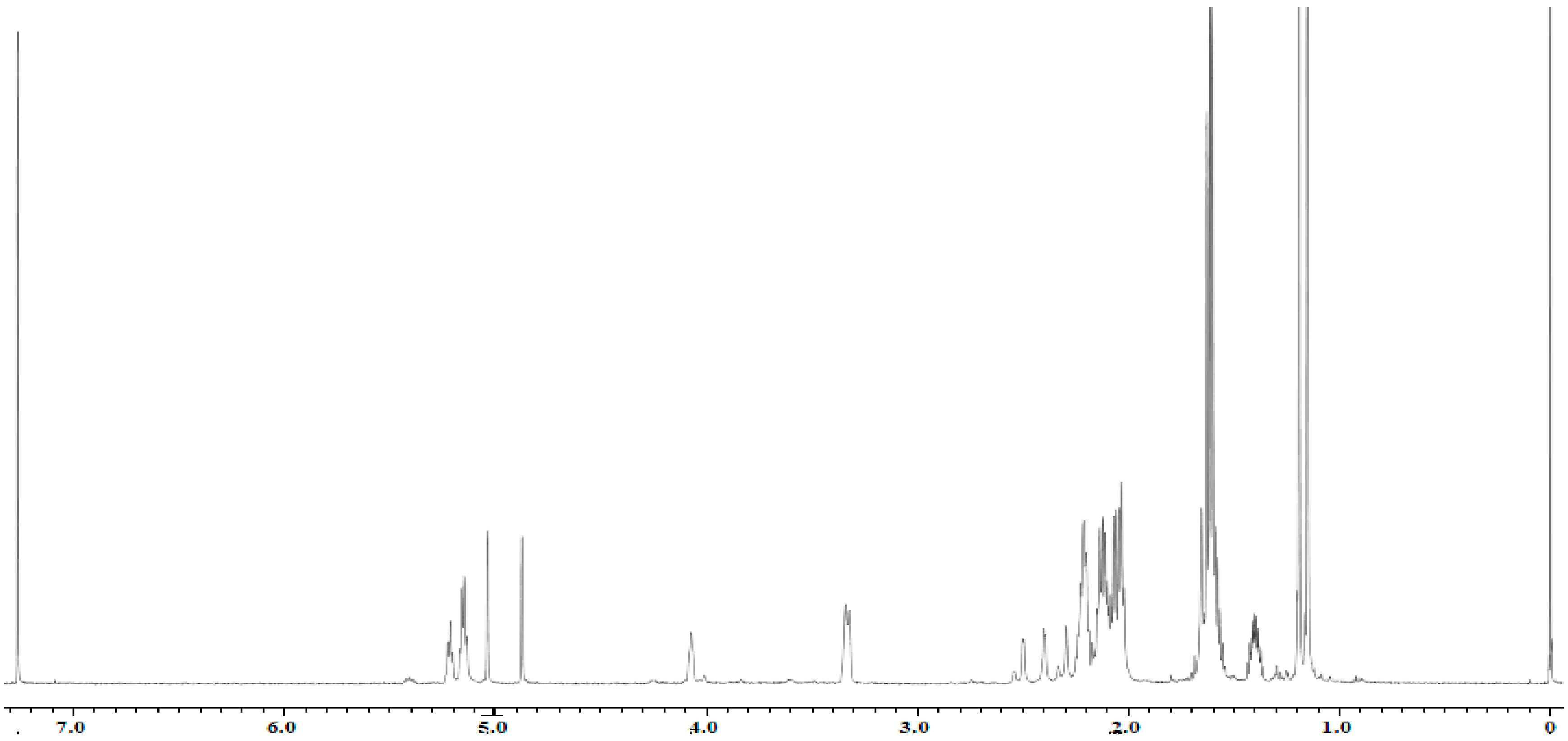

Figure S27. ^1H NMR spectrum of 2,3,6,22,23-pentahydroxy-2,10,15,19,23-hexamethyl-7-methylenetetracosa-10,14,18-triene (**4**) (600 MHz in $CDCl_3$).

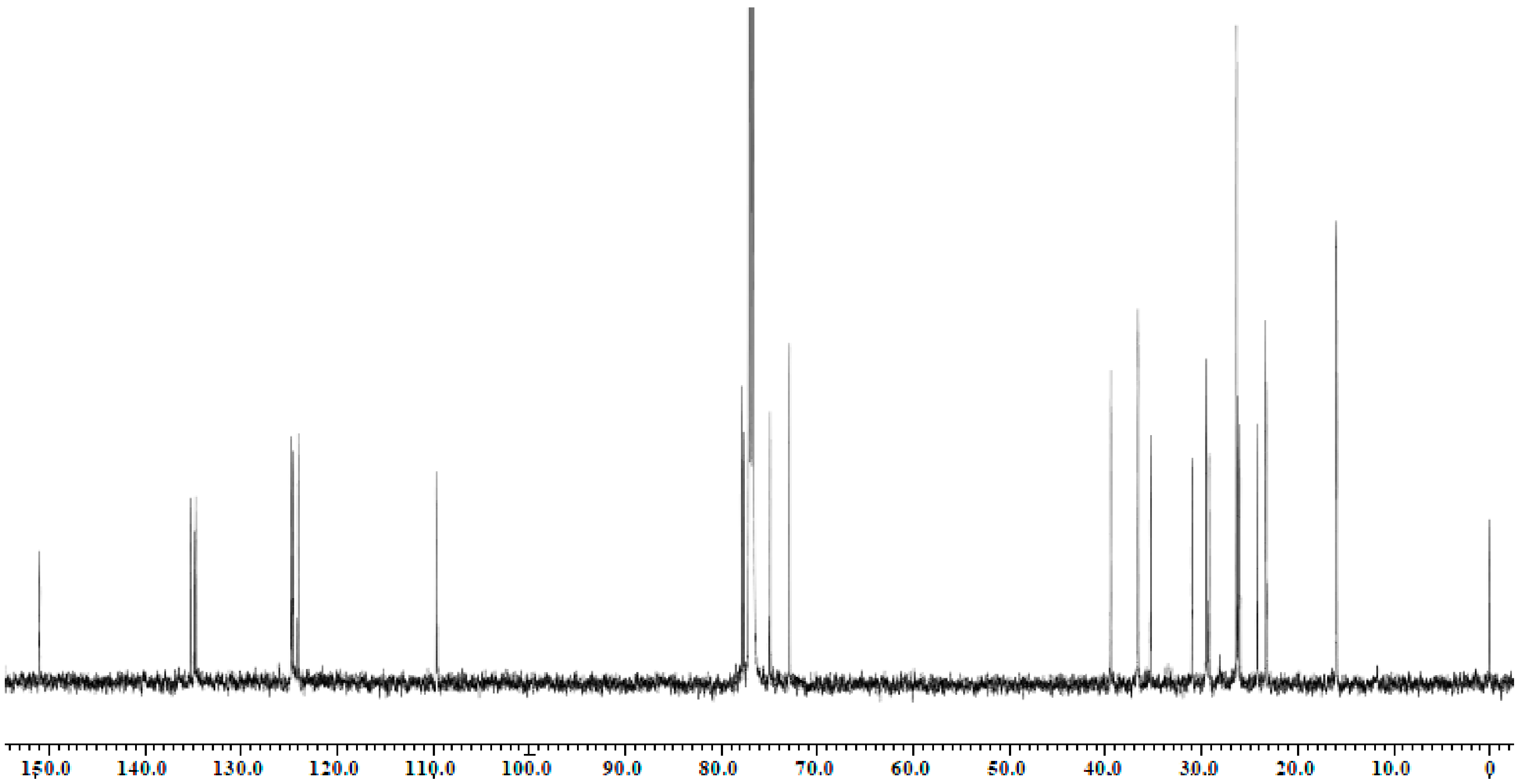

Figure S28. ^{13}C NMR spectrum of 2,3,6,22,23-pentahydroxy-2,10,15,19,23-hexamethyl-7-methylenetetracosa-10,14,18-triene (**4**) (150 MHz in $CDCl_3$).

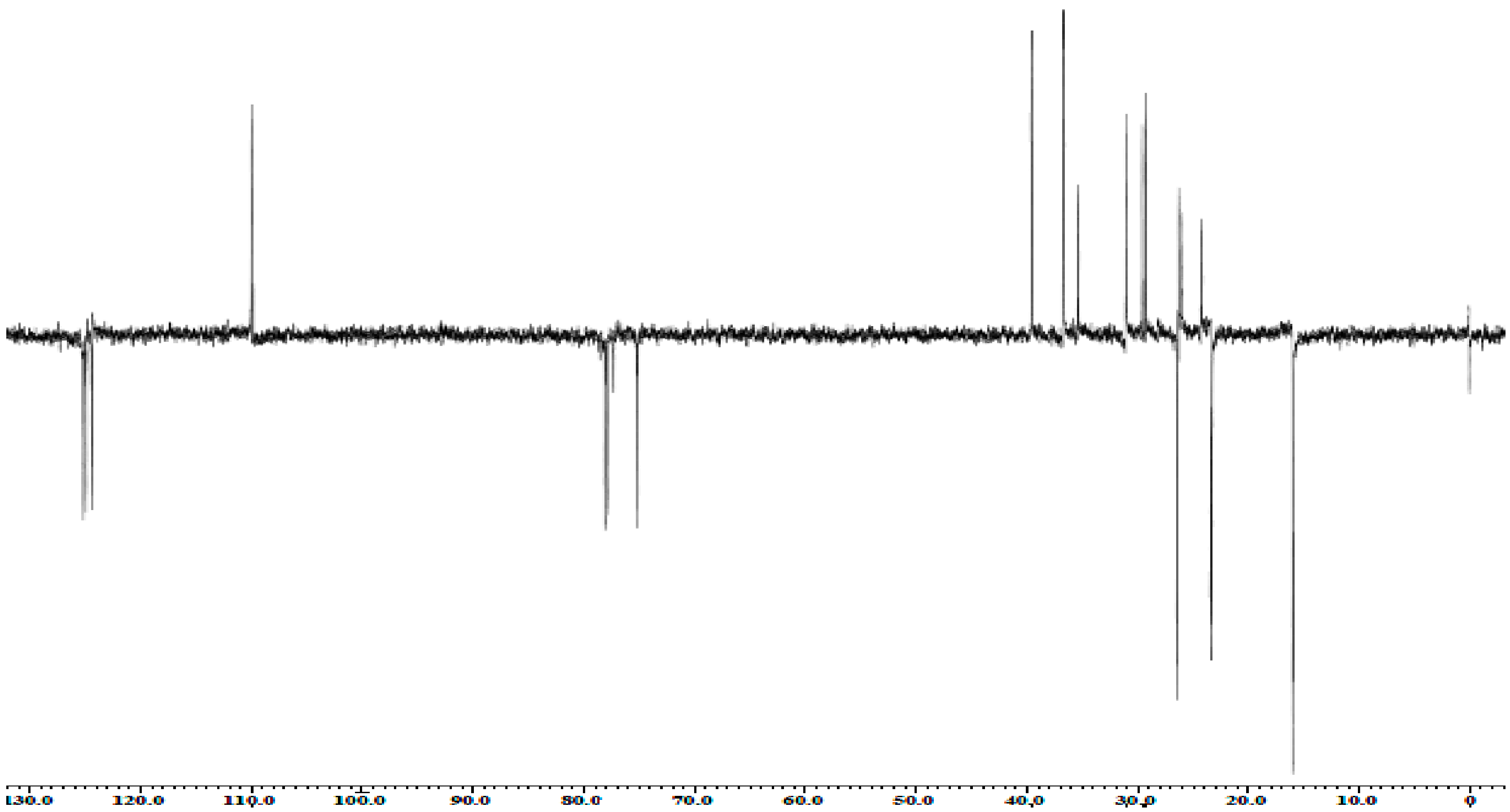

Figure S29. DEPT-135 NMR spectrum of 2,3,6,22,23-pentahydroxy-2,10,15,19,23-hexamethyl-7-methylenetetracosa-10,14,18-triene (**4**) (150 MHz in $CDCl_3$).

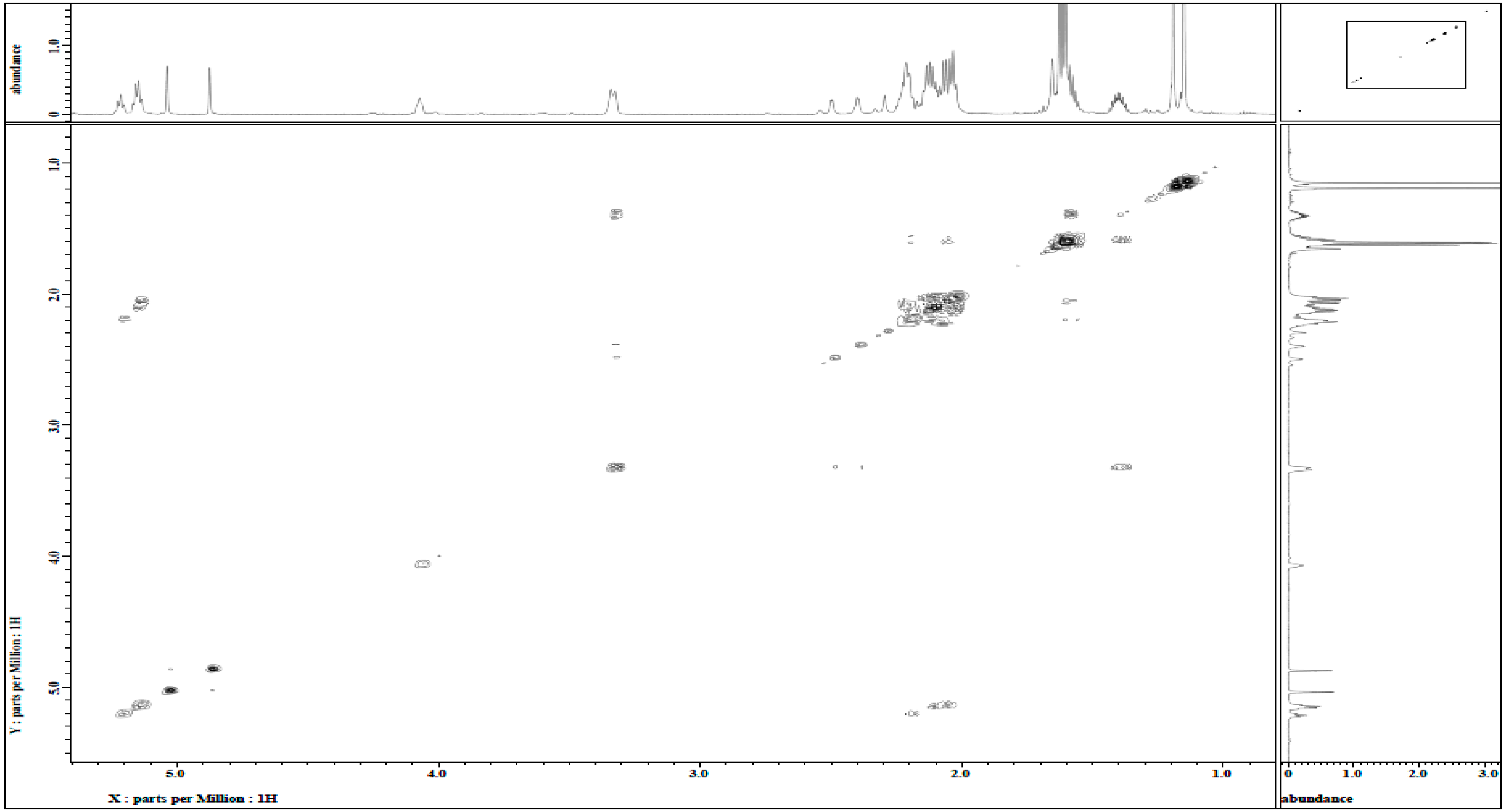

Figure S30. ^{1}H-^{1}H COSY NMR spectrum of 2,3,6,22,23-pentahydroxy-2,10,15,19,23-hexamethyl-7-methylenetetracosa-10,14,18-triene (**4**).

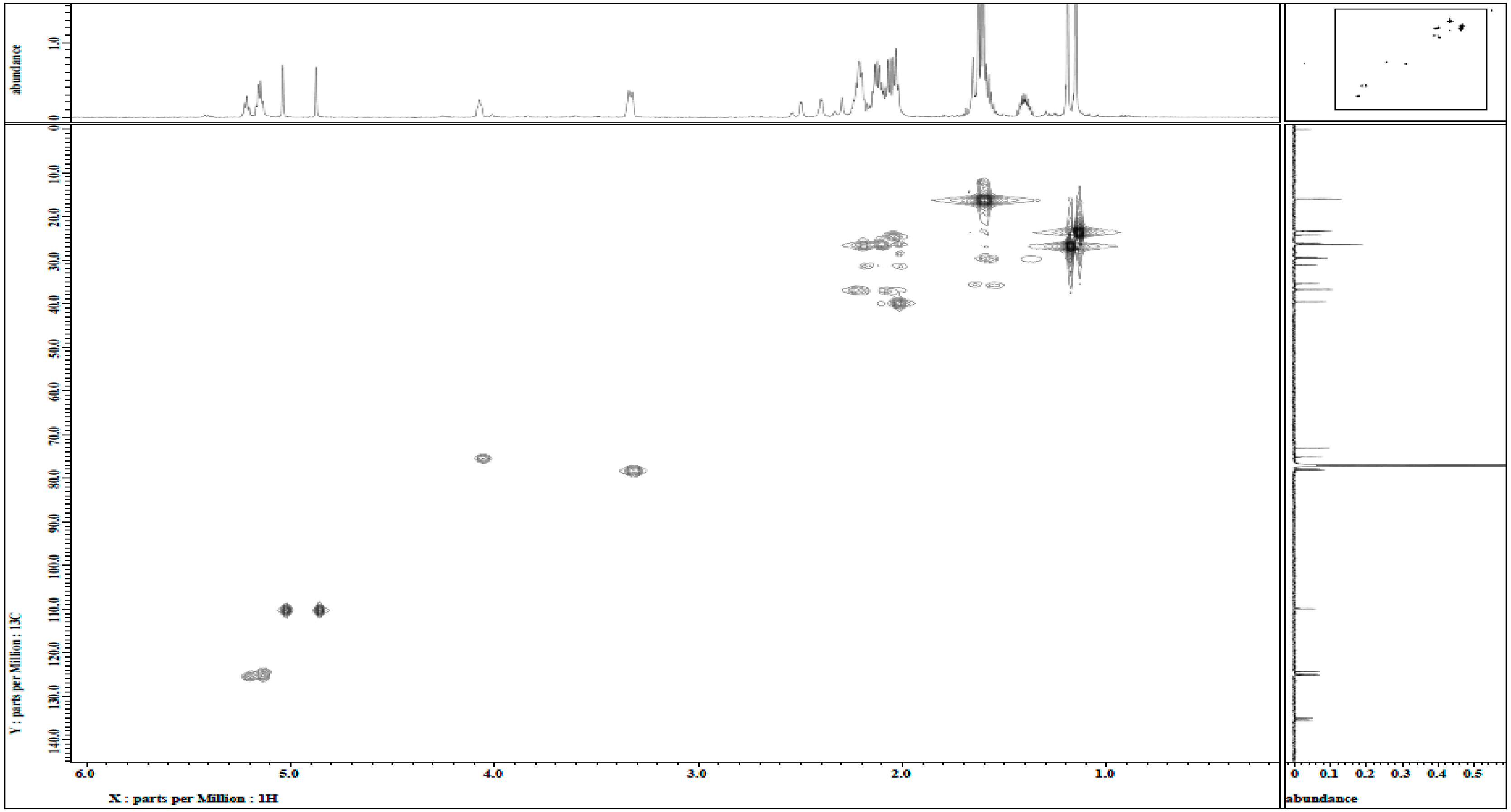

Figure S31. HMQC NMR spectrum of 2,3,6,22,23-pentahydroxy-2,10,15,19,23-hexamethyl-7-methylenetetracosa-10,14,18-triene (**4**).

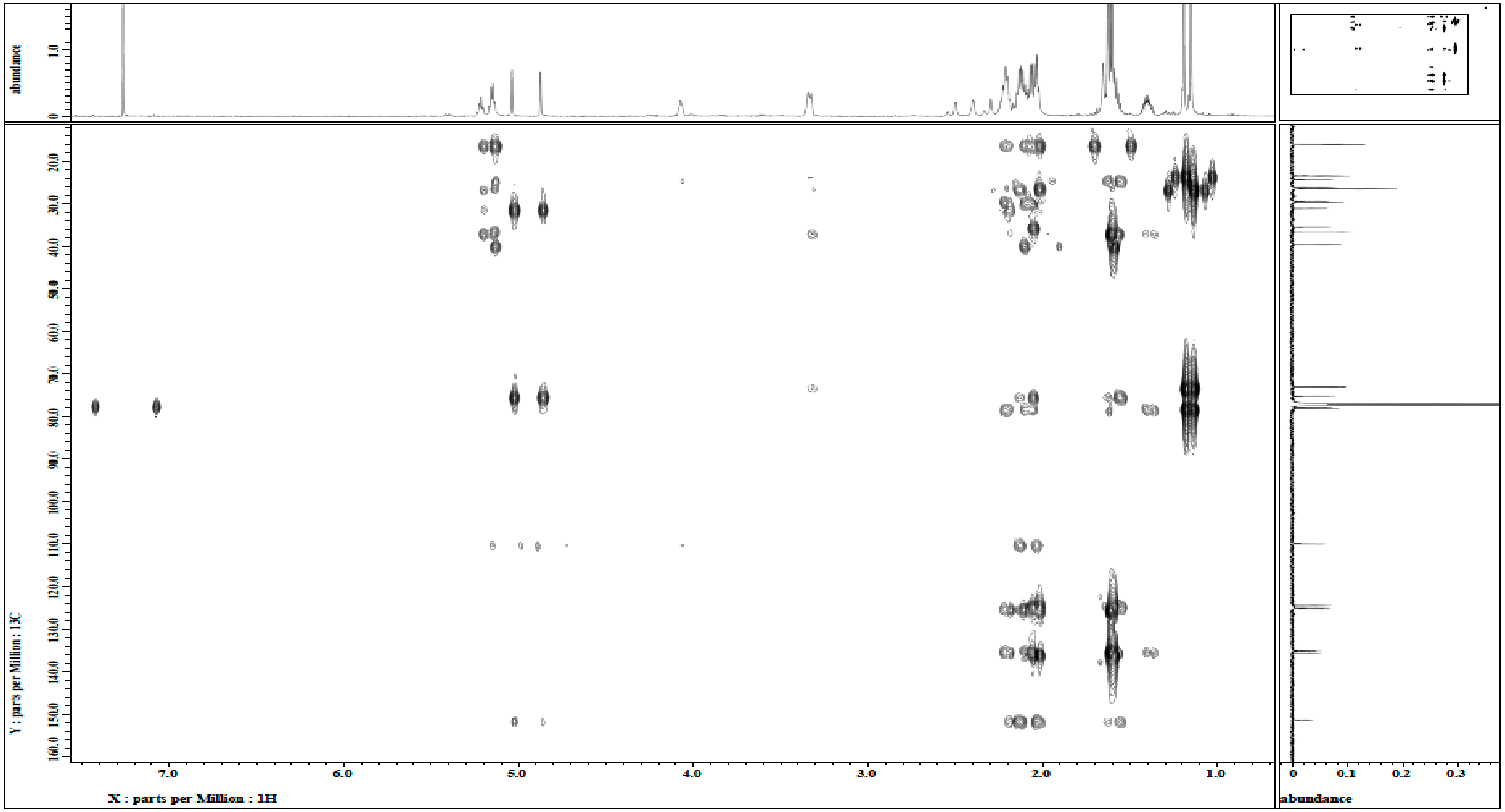

Figure S32. HMBC NMR spectrum of 2,3,6,22,23-pentahydroxy-2,10,15,19,23-hexamethyl-7-methylenetetracosa-10,14,18-triene (**4**).

Data File: D:₩ESI 기기지원 Data₩20111229₩CDG_5.lcd

Elmt	Val.	Min	Max
H	1	0	300
C	4	0	150
N	3	0	0

Elmt	Val.	Min	Max
O	2	0	12

Use Adduct
H

Error Margin (ppm): 10
HC Ratio: unlimited
Max Isotopes: all
MSn Iso RI (%): 75.00

DBE Range: not fixed
Apply N Rule: yes
Isotope RI (%): 1.00
MSn Logic Mode: AND

Electron Ions: both
Use MSn Info: no
Isotope Res: 10000
Max Results: 10

Event#: 2 MS(E-) Ret. Time : 1.829 Scan# : 466

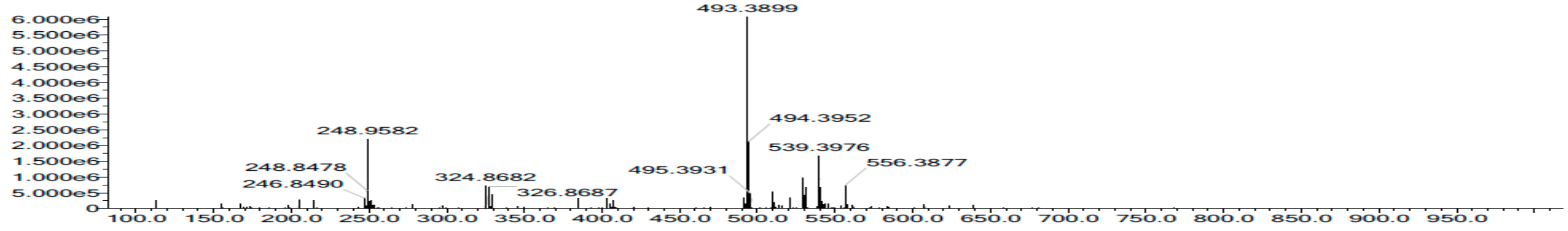

C30 H54 O5 [M-H] - : Predicted region for 493.3898 m/z

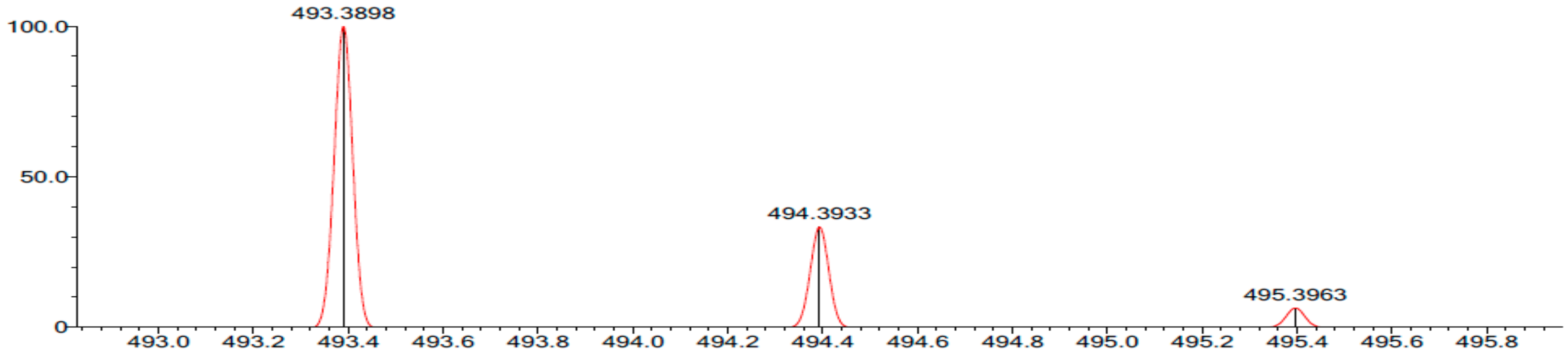

Rank	Score	Formula (M)	Ion	Meas. m/z	Pred. m/z	Df. (mDa)	Df. (ppm)	Iso	DBE
1	88.71	C30 H54 O5	[M-H] -	493.3899	493.3898	0.1	0.20	88.71	4.0

Figure S33. HR-ESI MS spectrum of 2,3,6,22,23-pentahydroxy-2,10,15,19,23-hexamethyl-7-methylenetetracosa-10,14,18-triene (**4**).

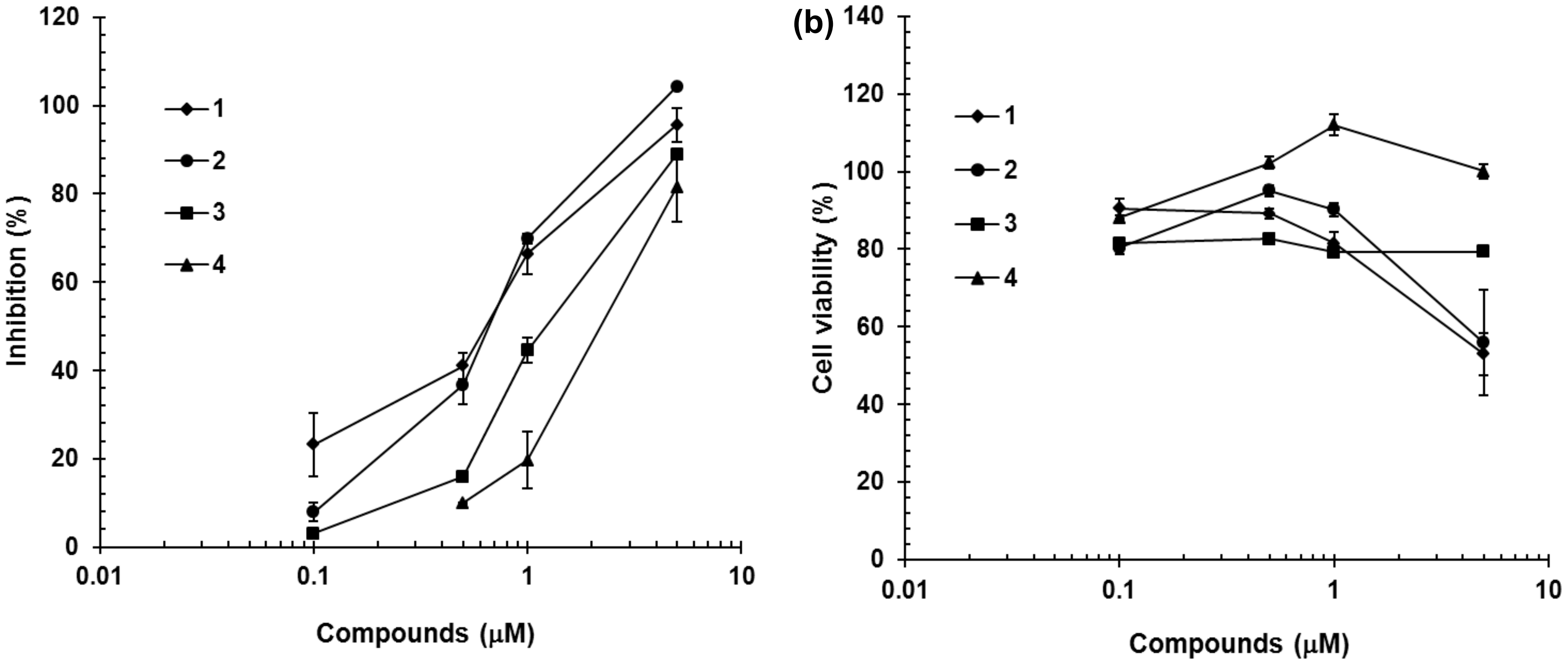

Figure S34. Inhibitory effects of compounds **2**-**4** on IL-6/STAT3 activation (a) and cell viability (b) in Hep3B cells.

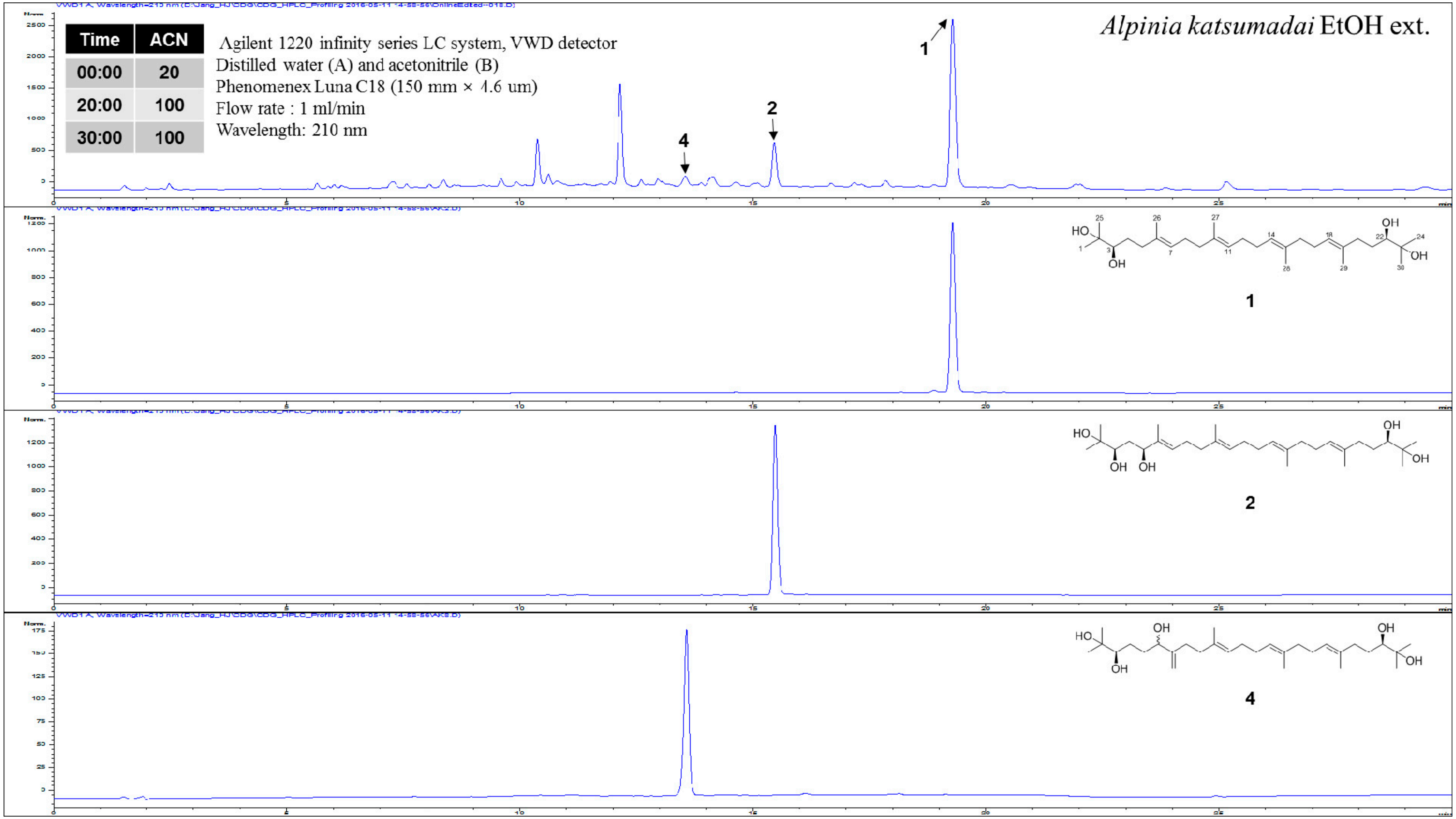

Figure S35. HPLC chromatogram of compounds **1**, **2**, and **4** of *A. katsumadai* ethanol-soluble extract (Agilent 1220 Infinity series; wavelength: 210 nm; column: Phenomenex Luna C_{18}; mobile phase: H_2O and acetonitrile; time of analysis: 30 min).

Table S1. Inhibitory effects of EtOH extract, $CHCl_3$ and H_2O layer of *A. katsumadai* on IL-6-induced STAT3 activation.

Samples (μg/mL)		Inhibition (%) [a]	Cytotoxicity (%) [a]
EtOH extract	0.5	45.3 ± 0.9	97.2 ± 0.8
	1	66.8 ± 3.6	101.4 ± 0.2
	5	102.7 ± 0.6	55.4 ± 1.6
$CHCl_3$ layer	0.5	29.2 ± 1.9	90.6 ± 0.1
	1	42.7 ± 2.9	92.0 ± 0.4
	5	90.0 ± 0.1	95.6 ± 0.4
H_2O layer	0.5	17.4 ± 4.5	87.5 ± 0.4
	1	19.6 ± 1.4	86.1 ± 0.5
	5	50.3 ± 4.6	93.2 ± 0.7

[a] The data are presented as the means from three independent experiments performed in duplicate.
